# The Plasmodesmal Protein PDLP1 Localises to Haustoria-Associated Membranes during Downy Mildew Infection and Regulates Callose Deposition

**DOI:** 10.1371/journal.ppat.1004496

**Published:** 2014-11-13

**Authors:** Marie-Cécile Caillaud, Lennart Wirthmueller, Jan Sklenar, Kim Findlay, Sophie J. M. Piquerez, Alexandra M. E. Jones, Silke Robatzek, Jonathan D. G. Jones, Christine Faulkner

**Affiliations:** 1 The Sainsbury Laboratory, Norwich Research Park, Norwich, United Kingdom; 2 John Innes Centre, Norwich Research Park, Norwich, United Kingdom; 3 Biological and Biomedical Sciences, Oxford Brookes University, Oxford, United Kingdom; 4 School of Life Sciences, University of Warwick, Coventry, United Kingdom; Scottish Crop Research Institute, United Kingdom

## Abstract

The downy mildew pathogen *Hyaloperonospora arabidopsidis* (*Hpa*) is a filamentous oomycete that invades plant cells via sophisticated but poorly understood structures called haustoria. Haustoria are separated from the host cell cytoplasm and surrounded by an extrahaustorial membrane (EHM) of unknown origin. In some interactions, including *Hpa*-Arabidopsis, haustoria are progressively encased by host-derived, callose-rich materials but the molecular mechanisms by which callose accumulates around haustoria remain unclear. Here, we report that *PLASMODESMATA-LOCATED PROTEIN 1* (*PDLP1*) is expressed at high levels in *Hpa* infected cells. Unlike other plasma membrane proteins, which are often excluded from the EHM, PDLP1 is located at the EHM in *Hpa*-infected cells prior to encasement. The transmembrane domain and cytoplasmic tail of PDLP1 are sufficient to convey this localization. PDLP1 also associates with the developing encasement but this association is lost when encasements are fully mature. We found that the *pdlp1,2,3* triple mutant is more susceptible to *Hpa* while overexpression of *PDLP1* enhances plant resistance, suggesting that PDLPs enhance basal immunity against *Hpa.* Haustorial encasements are depleted in callose in *pdlp1,2,3* mutant plants whereas *PDLP1* over-expression elevates callose deposition around haustoria and across the cell surface. These data indicate that PDLPs contribute to callose encasement of *Hpa* haustoria and suggests that the deposition of callose at haustoria may involve similar mechanisms to callose deposition at plasmodesmata.

## Author Summary

Haustoria are specialised invasive structures that project from fungal or oomycete hyphae into host plant cells during infection, acting as sites for molecular exchange between host and pathogen. Haustoria are targets of plant defence responses, including the deposition of membranes and polysaccharides in an encasement structure that surrounds the haustorium. It is assumed that the encasement physically seals the haustorium off from the host cell. Here we have used cell biological and genetic approaches to reveal that the plasmodesmata-associated receptor-like protein PDLP1 plays a role in infection success of the Arabidopsis downy mildew pathogen, specifically in the development of the encasement. Using live cell imaging, we observed that PDLP1 relocates to the extra-haustorial membrane, and this is required for deposition of the polysaccharide callose in the encasement. This directly correlates pathogen success with the structure of the encasement, verifying the significance of the encasement in host defence. Further, our data pose the possibility that callose deposition at plasmodesmata and the haustorial encasement exploit similar mechanisms. Our findings shed light on plant defences at haustoria and how they inhibit pathogen success.

## Introduction

Eukaryotic filamentous pathogens such as rusts, powdery mildew fungi, and oomycetes including Arabidopsis downy mildew *Hyaloperanospora arabidopsidis* (*Hpa*) and *Phytophthora spp*., form specialized feeding structures in host cells called haustoria. Haustoria are unicellular protrusions from hyphae and function as the site of molecular exchange of nutrients and effectors between host and pathogen [1]. In the model interaction between the biotrophic pathogen *Hpa* and its natural host Arabidopsis, this invasive process induces subcellular rearrangements in host cells, particularly in the membranes surrounding the invasive structure [2]–[4]. For fungi and oomycetes, haustoria present a host-pathogen interface in which the pathogen is separated from the host cytoplasm by different layers: the extrahaustorial matrix (EHMx) which contains cell wall material derived from the pathogen and the plant, and the host extrahaustorial membrane (EHM) [5]–[7]. The EHM is continuous with the host plasma membrane (PM) but differs in protein composition [8]–[10] and appearance [6], [11], [12] suggesting functional specialisation of this membrane domain. During fungal infection, the EHM and PM at the site of invasion may be constricted by one or more neck bands [5], [13], [14] physically sealing the EHMx off from the host cell wall. Analogous but less densely stained structures have been observed in oomycete – plant interactions [15].

After successful entry in the host tissue, plant pathogens often encounter post-invasive defence barriers, such as depositions of host-derived material at haustoria. These materials include membranes, callose, cellulose, pectin, silicon, phenolic compounds, antimicrobial peptides, toxic secondary metabolites and reactive oxygen species [16]–[23], and following initial deposition at the neck of haustoria progressively encase the entire structure [2], [24]. Callose deposition is considered as a hallmark of plant defence responses [25] but the direct role of callose deposition in defence against an oomycete pathogen has not yet been determined.

Phospholipid membranes define cellular and subcellular structures. In eukaryotic cells, the PM is the outermost of the cellular membranes, encasing the cytoplasm and cellular organelles. The PM is not uniform in composition but contains specialised domains that may perform different functions. Indeed, it has recently been shown in plant cells that the protein composition of membrane domains changes following elicitation with pathogen-associated molecular patterns (PAMPs) [26], and that different receptor complexes form in different membrane domains [27] suggesting that protein activation can be confined to specific membrane domains. Plasmodesmata (PD) are PM lined channels that bridge plant cell walls, creating membrane and cytoplasmic continuity between adjacent cells. The PM that lines these pores is proposed to be a specialised PM domain [28] and this membrane has been found to contain functionally specialised receptors [27], [29], Remorin [30] (specific to lipid rafts) and TETRASPANIN3 [31] (associated with tetraspanin enriched microdomains). The identity of proteins that are present and function at PD is poorly characterised [32] and while the functional significance of the proteinaceous composition of the membranes within PD is not fully understood, membrane specialisation is assumed to relate to the regulation of molecular flux between cells [32]. Recently, a number of membrane proteins have been identified as PD-located but it is unclear how these proteins are specifically recruited to this membrane domain.

The PD LOCATED PROTEIN (PDLP) family is composed of eight receptor-like proteins which contain a cytoplasmic domain, a single transmembrane domain and two extracellular Domains of Unknown Function 26 (DUF26) [33]. PDLPs are recruited to PD membranes via their transmembrane domain [33], where they are exploited as a scaffold or receptor for viral movement proteins for the assembly of viral tubules through PD [34]. It has been noted that PDLPs exhibit functional redundancy, as might be expected for members of a gene family with overlapping patterns of expression [35]. PDLP5 was recently identified as a mediator of salicylic acid (SA) induced PD closure, a process required for resistance against the bacterial pathogen *Pseudomonas syringae* pv. *maculicola* [36]. PDLP5 activity is correlated with callose deposition at PD [36], which induces PD closure [37]. PDLPs have also been associated with the transmission of herbivory responses [38]. However, despite these clues to their functional context, the molecular function of PDLPs has still not been identified.

In this study we found that in addition to its PD-associated function, PDLP1 mediates callose deposition around *Hpa* haustoria and that this activity is required for plant immunity. *PDLP1* expression is specifically upregulated in mesophyll cells harbouring *Hpa* haustoria and PDLP1-GFP localises at the EHM of developing haustoria prior to encasement where, when overexpressed, it promotes EHM membrane proliferation. PDLPs are required for callose encasement of the haustoria and this is negatively correlated with infection success. These data suggest that PDLPs are involved in callose deposition at multiple cellular locations that include PD and haustoria.

## Results

### *PDLP1* is upregulated in haustoria-containing cells

**Figure 1:**
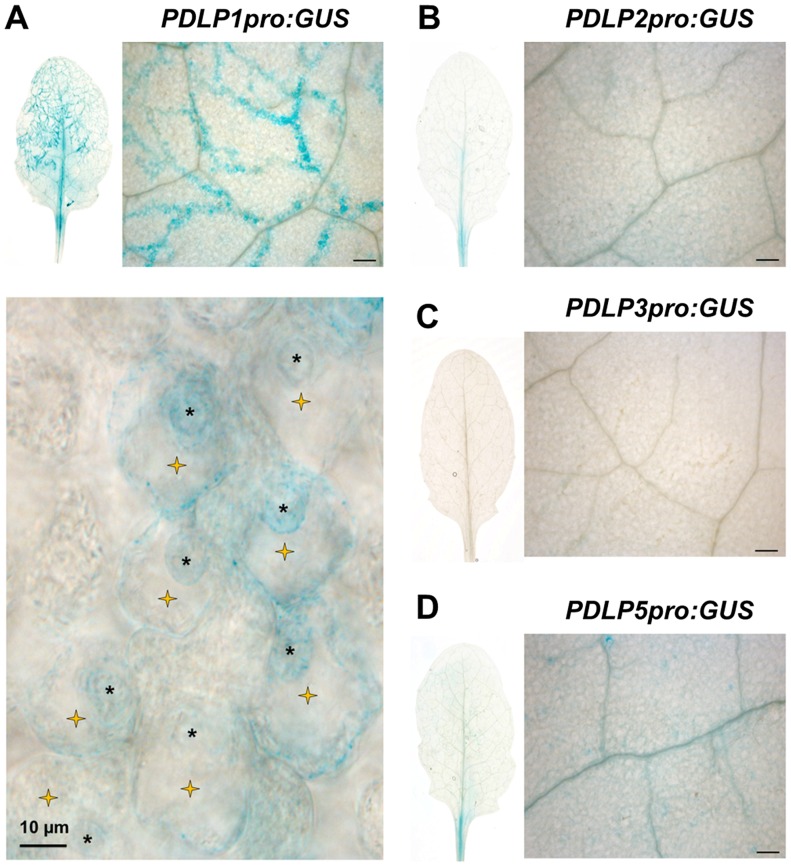
PDLP1 is specifically expressed in *Hpa* infected mesophyll cells. GUS staining of *PDLP1pro:GUS* (A), *PDLP2pro:GUS* (B), *PDLP3pro:GUS* (C), *PDLP5pro:GUS* (D) in *Arabidopsis* leaves 6 days post inoculation with *Hpa* Waco9 shows that GUS staining correlates with *Hpa* growth in *PDLP1pro:GUS* expressing plants. At higher magnification, GUS staining is restricted to cells harbouring haustoria (yellow stars) while no GUS staining was detected in non-infected mesophyll cells. Haustoria are indicated by asterisks. Scale bars are 200 µm unless otherwise indicated.

*PDLP5* transcriptionally responds to SA and has a role in defence against hemibiotrophic bacteria [36]. To assess if other members of the PDLP family are expressed in response to pathogen inoculation, we first checked the expression pattern of the eight *PDLP* genes during a time course of *Hpa* Waco9 infection in Arabidopsis Col-0. Using recently available transcriptomic data [39], we observed that both *PDLP1* and *PDLP5* expression was increased 5 days post inoculation (DPI) when compared to 3 DPI (Figure S1). To determine if PDLP1 plays a role in defence we examined expression of both *PDLP1* and *PDLP5* at the cellular level during *Hpa* infection. Plants stably expressing promoter::GUS fusions were generated for *PDLP1* and *PDLP5* and examined for GUS expression 5 DPI. As negative controls, we compared *PDLP1* and *PDLP5* expression to *PDLP2* and *PDLP3* using plants expressing *PDLP2pro:GUS* and *PDLP3pro:GUS* [35]. While some low level expression was evident for *PDLP2* and *PDLP5*, neither the *PDLP2*, *3* nor *5* promoters showed *GUS* expression that was associated specifically with *Hpa* infection (Figure 1). By contrast, GUS staining for *PDLP1pro:GUS* was visible in cells harbouring haustoria along *Hpa* hyphae (Figure 1). This result indicates that in contrast to *PDLP5*, the *PDLP1* promoter is upregulated specifically at the site of *Hpa* cellular invasion.

### PDLP1 locates at the EHM during early time points of *Hpa* infection

**Figure 2:**
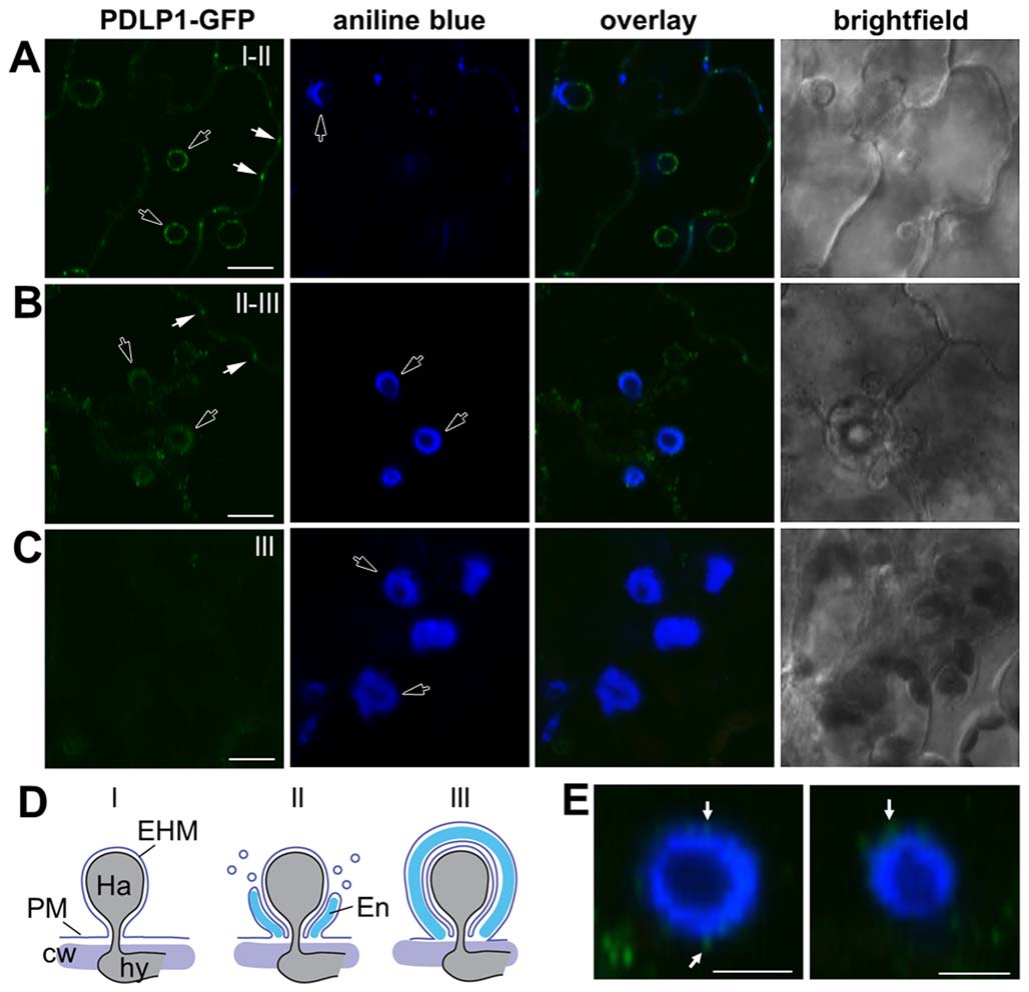
PDLPs localise to the extra-haustorial membrane. PDLP1-GFP is observed at the EHM prior to encasement. (A) In unencased haustoria, or those with a developing encasement at the haustorial neck, PDLP1-GFP is present in the EHM surround the haustorium (n = 50/50). PD-located signal is indicated with solid arrows, while open arrows indicate signal associated with haustoria. (B) As the encasement develops (stained with aniline blue, open arrows) and surrounds the entire haustorium PDLP1-GFP fluorescence remains associated with the haustorium and PDLP1-GFP positive bodies can be seen at the encasement periphery (see E). (C) When encasements are mature PDLP1-GFP is no longer associated with the structure. (D) Scheme of the development of the *Hpa* haustorial encasement, stages I, II and III are indicated in (A–C) for reference. (E) Enlargement of developing encasements shows PDLP1-GFP positive bodies at the periphery of the encasement (arrows). Scale bars are 20 µm (A–C) and 10 µm (E).

**Figure 3:**
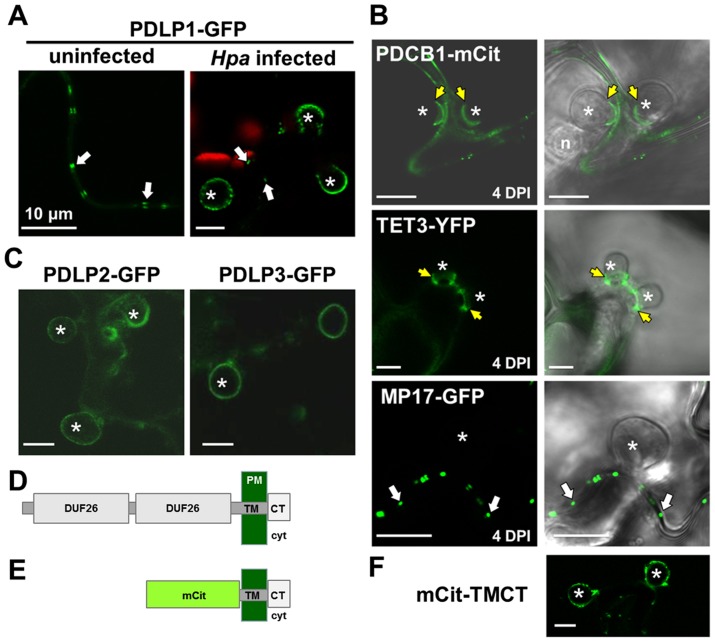
PDLPs, but not other PD proteins, are located at the EHM. (A) PDLP1-GFP is located at PD (arrows) in both uninfected and infected tissue. During infection, PDLP1-GFP is also located at haustoria (right). (B) EHM association is specific to PDLPs as the PD markers PCBD1-mCit, TET3-YFP and MP17-GFP do not locate to the EHM. PDCB1-mCit and TET3-YFP locate to the developing encasement (n = 13/13 and n = 12/12 respectively) and MP17-GFP shows no association with haustoria (n = 5/5). Images are fluorescent data (left) and fluorescence/transmitted light overlays (right). (C) PDLP2-GFP and PDLP3-GFP are located at the haustoria periphery (n = 10/10 and n = 5/17 respectively). (D) and (E) Diagrammatic representation of the topology of the PDLP1 and synthetic mCit-TMCT proteins following cleavage of the signal peptide. PDLP1 has 2 extracellular DUF26 domains, the transmembrane (TM) domain and cytoplasmic tail (CT), which projects into the cell cytoplasm (cyt). In the mCit-TMCT variant, the TM and CT is fused to mCitrine. (F) mCit-TMCT localises to the EHM similar to PDLP1-GFP (n = 10/10). Asterisks, haustoria; yellow arrows, developing encasement; white arrows, PD. Scale bars are 10 µm.

Given that *PDLP1* is specifically expressed in haustoria-containing cells we examined the subcellular location of PDLP1-GFP after *Hpa* infection to determine if this increase in expression is likely to affect PD function. Plants that constitutively express *PDLP1-GFP* under the *35S* promoter (PDLP1 OE, [33]) were imaged at 3–6 DPI to observe haustoria at various stages of encasement (Figure 2). In uninfected leaves, PDLP1-GFP localises to PD (white arrows, Figure 3A, [33]). Following inoculation with *Hpa* PDLP1-GFP was visible in the PD and surrounding unencased haustoria (Figure 2A, 3A). Infiltration of infected tissue with aniline blue stained any developing, callose-filled encasements. Haustoria with developing encasements showed aniline blue staining at the neck of the haustorium while PDLP1-GFP completely surrounded the structure (Figure 2A), illustrating that PDLP1-GFP associates with haustoria prior to development of the encasement. PDLP1-GFP remained associated with the haustorium as the encasement developed (Figure 2B) but was not associated with fully encased haustoria at a late stage of development (Figure 2C). During the encasement process, small PDLP1-GFP containing bodies could be seen peripheral to the haustorium (Figure 2E). These bodies are possibly secretory vesicles depositing encasement material at the developing structure. The localisation of the PDLP1-GFP fusion during infection was also imaged when expressed from its native promoter. In plants stably expressing *PDLP1pro::PDLP1-GFP* [33], PDLP1-GFP was observed surrounding haustoria (Figure S2A). Like in PDLP1 OE plants, PDLP1-GFP was also observed in the developing encasement, but sometimes this association with the encasement could be resolved into two layers that suggest PDLP1-GFP is concentrated in membranes surrounding the encasement (Figure S2B). Many PM proteins are not present in the EHM but are associated with the haustorial encasement, i.e. they are observed at the neck of haustoria early in encasement development and completely surrounding the haustorium when the encasement is fully developed [3]. Localisation of PDLP1 at haustorial membranes prior to encasement suggests it is differentially incorporated into the EHM relative to other PM proteins. Significantly, this localisation also indicates that PDLP1 has a non-PD associated function.

In order to establish whether or not PDLP localisation at haustoria is characteristic of PD proteins, we next examined the localisation of fusions to the PD-associated membrane proteins MOVEMENT PROTEIN-17 (MP17, Figure S3, [40]), TETRASPANIN3 (TET3; Figure S3 [31]) and the PD CALLOSE BINDING PROTEIN 1 (PDCB1, Figure S3, [41]) in unencased haustoria (Figure 3B). Each fusion was expressed from the *35S* promoter. As observed for other PM-localised proteins, PDCB1-mCit and TET3-YFP were both visible in the developing encasement (Figure 3B) while MP17-GFP showed no association with haustoria (Figure 3B). Since PD-associated proteins did not localise at the EHM during *Hpa* infection, we concluded that the haustorial association is specific to PDLPs. Indeed, similar to PDLP1-GFP, PDLP2-GFP and PDLP3-GFP, from *35S* promoter expression, were also observed surrounding unencased haustoria (Figure 3C, S3). Qualitative assessment of these images indicates that fluorescence associated with haustoria is fainter for these marker proteins, and when combined with the observation that PDLP3-GFP was not always visible at the haustorial periphery, raises the possibility these PDLPs have a weaker association with haustorial membranes. Irrespective, this observation indicates that, while not expressed at high levels in haustoria-containing cells, other PDLP family members carry targeting information for haustorial structures.

PDLPs have two extracellular DUF26 domains, a transmembrane (TM) domain and a short cytoplasmic tail (CT) (Figure 3D, [33]). A construct that fuses the fluorescent protein mCitrine (mCit) between the signal peptide and C-terminus (including the transmembrane domain and cytoplasmic tail) of PDLP1 (mCit-TMCT, Figure 3E) targets mCitrine to PD [33]. To determine if haustorial targeting information is also contained within the C-terminal domains of the protein, we examined the localisation of mCit-TMCT during *Hpa* infection. As found for PDLP1-GFP, mCit-TMCT is located surrounding unencased haustoria (Figure 3F). Thus, PDLP targeting to haustoria is conferred by the PDLP1 C-terminal tail and/or the transmembrane domain.

### PDLPs are required for resistance against *Hpa*

**Figure 4:**
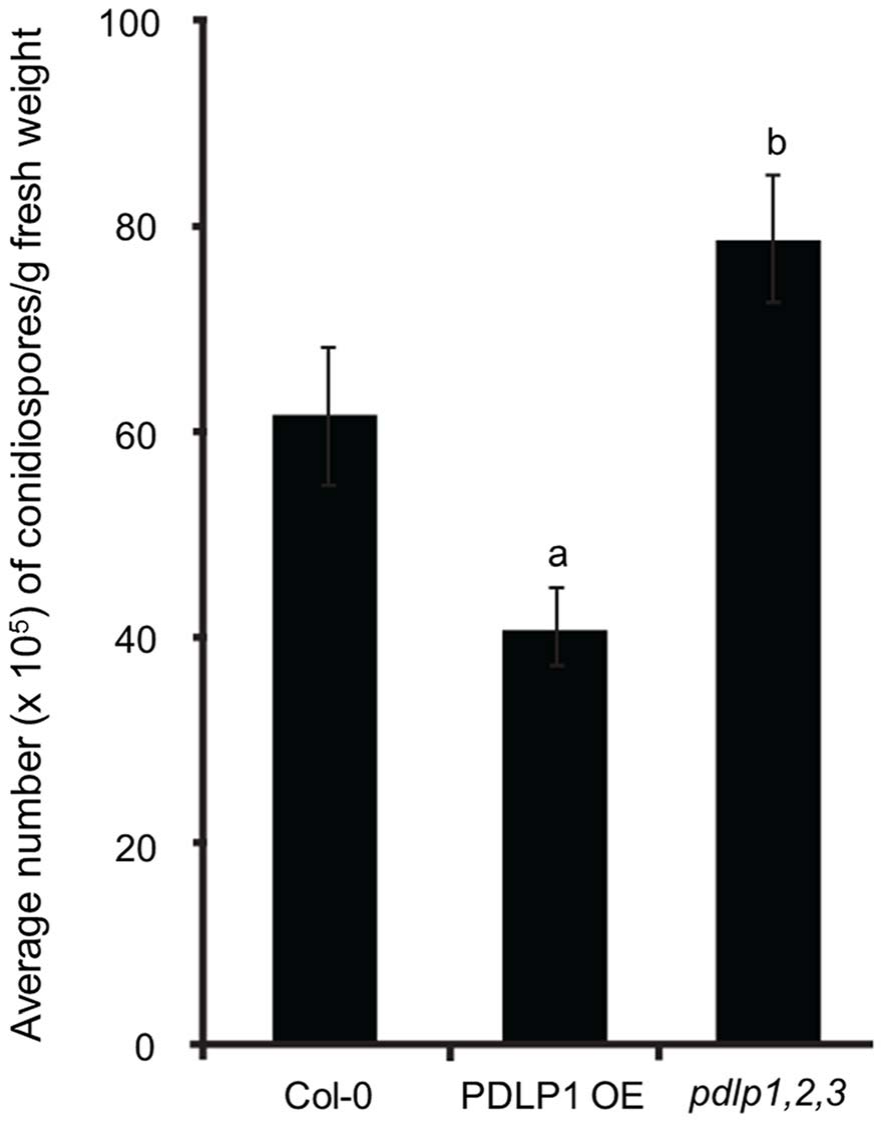
PDLPs are required for plant immunity against *Hpa*. *Hpa* Waco9 sporulation 5 DPI is reduced in PDLP1 OE plants but increased in *pdlp1,2,3* mutant plants when compared to wild type Col-0. Error bars represent the standard error of the mean. a and b denote statistical significance (p-value <0.05) when the data is analysed by one-way ANOVA and a Tukey-Kramer test.

To test whether PDLPs play a role in defence against *Hpa*, we assessed *Hpa* susceptibility in transgenic and mutant lines. Expression of *35S::PDLP1-GFP* (PDLP1 OE) significantly impairs molecular flux between leaf epidermal cells but a *pdlp1* knockout mutant showed no alterations in molecular flux compared with Col-0 [33]. However, double knockout mutants for *pdlp1,2* and *pdlp2,3* showed increased molecular flux suggesting functional redundancy within the protein family [33]. For this reason, the triple knockout mutant *pdlp1,2,3* [38] was used in all mutant assays. Following spray inoculation with the compatible isolate *Hpa* Waco9, *Hpa* sporulation 6 DPI was reduced in PDLP1 OE relative to wild-type Col-0 plants while *Hpa* sporulation was increased in *pdlp1,2,3* mutant plants (Figure 4). These results indicate that PDLP1 is a positive regulator of plant immunity against *Hpa*.

While Arabidopsis Col-0 ecotype exhibits a compatible interaction with *Hpa* Noco2, *Hpa* isolate Emoy2 is recognised by the Resistance (R)-protein RPP4 in Col-0 [42]. To determine whether PDLPs play a role in RPP4-mediated resistance, we assayed the *pdlp1,2,3* mutant for changes in susceptibility toward *Hpa* Emoy2. A small but significant increase in the number of conidiophores on *pdlp1,2,3* mutants relative to Col-0 was observed suggesting that PDLPs also positively regulate immunity in response to Emoy2 (Figure S4). *pdlp1,2,3* mutants exhibit a two-fold increase in conidiophore development relative to Col-0 while *rpp4* mutants exhibit a 35-fold increase in conidiophore development [43]. Given the haustorial location of PDLP1 it seems unlikely that it would act downstream of cytoplasmic RPP4, and more likely that PDLPs positively regulate a basal defence response.

### PDLP1 co-immunoprecipitates with the SNARE VAMP721

To identify other resident proteins of PDLP1-containing membranes, we immuno-purified (IP) PDLP1-GFP from both infected and uninfected tissues. Proteins that co-immunoprecipitate with PDLP1 (Table 1) were classified as proteins identified in PDLP1 OE samples only, i.e. absent from control samples, or those for which the ratio of spectrum counts for PDLP1 OE (infected or non-infected): control was greater than or equal to 4. Further, candidates were restricted to those that are located in cellular membranes (based on GO Cellular Component terms; PM, endosomes, vesicles, tonoplast), or are associated with compartments known to be subcellular locations of PDLP1 (ER, Golgi, PD [33]). Tandem mass spectrometry identified an almost identical subset of proteins in both infected and uninfected tissue samples (Table 1, Table S1). Several candidates have been implicated in plant defence, notably PEN3 [44], PEN1 [45], WAK2 [46], AHA1 [47] and VAMP721 [48], [49]. VAMP721 is implicated in delivery of the resistance protein RPW8 to the EHM during *Golovinomyces orontii* infection of Arabidopsis [48]. Others have functions associated with lipid modification, such as the phosphatidylinositol interactor PCAP1 [50], the phosphatidate phosphatase PAP1 [51], and the SEC14 domain protein PATL1 [52]. PATL1 [52] and VAMP721 [53], [54] are found at the cell plate which, like PD and haustoria, is another location at which callose is deposited.

**Table 1:** Proteins present in PDLP1-GFP containing membranes identified by MS/MS.

			Spectrum Counts		
Database Match	Protein name or description	Subcellular location	control	PDLP1 OE	PDLP1 OE *Hpa*
AT1G04750	VESICLE-ASSOCIATED MEMBRANE PROTEIN 721 (VAMP721)	CP, E, PM, PD	1	7	3
AT1G17840	WHITE-BROWN COMPLEX HOMOLOG PROTEIN 11 (WBC11)	PM	0	2	2
AT1G20200	EMBRYO DEFECTIVE 2719 (EMB2719), HAPLESS 15 (HAP15)	PM, PD	0	2	4
AT1G21270	WALL-ASSOCIATED KINASE 2 (WAK2)	PM	0	1	2
AT1G47128	RESPONSIVE TO DEHYDRATION 21 (RD21)	CW, PD	1	6	2
AT1G51500	WHITE-BROWN COMPLEX HOMOLOG PROTEIN 12 (WBC12)	PM	0	1	4
AT1G57720	translation elongation factor EF1B	CW, PM, PD	1	5	1
AT1G59870	PENETRATION 3 (PEN3), PLEIOTROPIC DRUG RESISTANCE (PDR8)	PM	0	2	4
AT1G72150	PATELLIN 1 (PATL1)	CP	0	4	3
AT1G72370	40S RIBOSOMAL PROTEIN SA (P40)	PM, PD	1	4	1
AT2G01180	LIPID PHOSPHATE PHOSPHATASE 1 (ATPAP1)	PM	0	4	5
AT2G18020	EMBRYO DEFECTIVE 2296 (EMB2296), ribosomal protein L4	PM, TP	0	4	2
AT2G18960	H(+)-ATPase 1 (AHA1)	PM	0	20	15
AT2G21390	Coatomer, alpha subunit	Ves, PD	0	7	2
AT2G45960	PLASMA MEMBRANE INTRINSIC PROTEIN 1B (PIP1;2)	PM	0	5	1
AT2G47610	Ribosomal protein L7Ae/L30e/S12e/Gadd45 family protein	PD, TP	0	3	1
AT3G03250	UDP-GLUCOSE PYROPHOSPHORYLASE 1 (UGP)	PM	0	2	1
AT3G09630	Ribosomal protein L4/L1	CW, PD, PM	0	3	3
AT3G11130	CLATHRIN HEAVY CHAIN 1 (CHC1)	Ves, PM, PD	0	5	0
AT3G11820	SYNTAXIN OF PLANTS 121 (SYP121), PENETRATION 1 (PEN1)	PM	0	1	2
AT3G14990	ATDJ1A, DJ-1 homolog,	PD, PM, TP	0	4	2
AT3G23400	FIBRILLIN 4 (FIB4)	PM	1	6	3
AT3G52880	MONODEHYDROASCORBATE REDUCTASE 1 (MDAR1)	CW, PM	0	4	1
AT4G11150	VACUOLAR ATP SYNTHASE SUBUNIT E1 (VHA-E1)	TP	0	3	0
AT4G28780	GDSL-like Lipase/Acylhydrolase superfamily protein	CW	0	1	2
AT5G12250	BETA-TUBULIN 6 (TUB6)	PM	1	2	5
AT5G14040	PHOSPHATE TRANSPORTER 3;1 (PHT3;1)	CW, M, TP	0	2	2
AT5G23860	BETA-TUBULIN 8 (TUB8)	M	1	7	5
AT5G26000	BETA GLUCOSIDASE 38 (BGLU38)	CW	1	1	5
AT5G28540	BiP (BIP1)	CW, ER, G, PD	2	14	12
AT5G43980	PDLP1	CW, PD	7	92	79
AT5G47210	mRNA binding family, AtVPS2.2-GFP Interacting Protein	PM	0	5	4
AT5G49360	BETA-XYLOSIDASE 1 (BXL1)	M, CW	0	4	1
AT5G61790	CALNEXIN 1 (CNX1)	ER, PD	0	1	5

Total spectrum counts for each protein are presented, these are summed unique spectrum counts from at least three replicate IPs. Full details of the proteins identified are presented in Table S1. Subcellular location based on GO cellular component terms (http://arabidopsis.org): cell plate (CP), endoplasmic reticulum (ER), endosomes (E), Golgi (G), plasma membrane (PM), plasmodesma (PD), tonoplast (TP), vesicles (Ves).

### PDLP1 regulates callose deposition in the developing encasement

**Figure 5:**
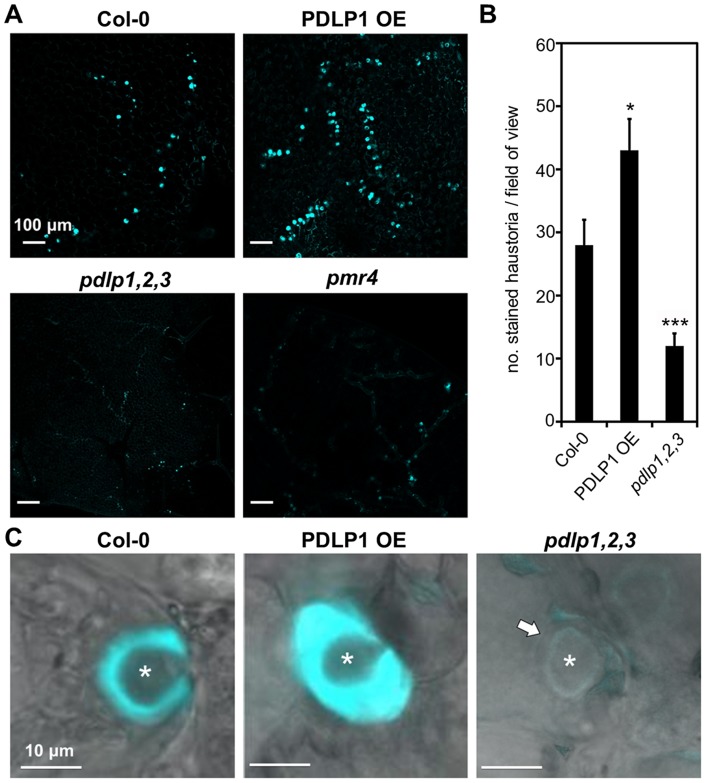
PDLP1 is required for callose deposition in haustorial encasement. (A) Aniline blue staining of callose in Col-0, PDLP OE, *pdlp1,2,3* and *pmr4* mutant leaves 5 DPI with *Hpa* Noco2 identifies that *pdlp1,2,3* produces fewer encased haustoria at this stage of infection, similar to the callose synthesis mutant *pmr4*. Quantification (B) of stained haustoria confirms that *pdlp1,2,3* produces significantly fewer aniline blue stained encasements than Col-0. PDLP1 OE plants produce more stained encasements than Col-0 plants per field of view. Error bars represent the standard error of the mean. * p-value <0.05, *** p-value <0.001 by Student's t-test. (C) At higher magnification, aniline blue stained encasements in PDLP1 OE cells appear thicker than Col-0 encasements. The transmitted light image of a *pdlp1,2,3* haustorium suggests that there is some encasement (arrow) of the haustorium but this structure does not stain with aniline blue. Asterisks indicate haustoria. Scale bars are 100 µm (A) and 10 µm (C).

Given that PDLP1-GFP is located at haustoria and PD, and that these membrane domains are both sites of callose deposition, we investigated whether PDLP1 plays a role in callose encasement of *Hpa* haustoria. Aniline blue staining of callose in infected leaves was used to assess callose deposition in encasements [21]. Staining of wild-type, PDLP1 OE and *pdlp1,2,3* leaves 4–5 DPI revealed differences in the frequency of haustorial encasements (Figure 5A). 4–5 DPI Col-0 and PDLP1 OE plants exhibited many haustoria fully encased in a callose-rich material (Figure 5A). By contrast, *Hpa* infected leaves of *pdlp1,2,3* plants showed few aniline-blue stained haustoria, similar to the callose synthase mutant *pmr4* (Figure 5A) [55]. We used automated callose detection [56] to quantify the number of aniline blue stained encasements in infected leaves. PDLP1 OE plants produced significantly more callose encasements per image area whereas *pdlp1,2,3* mutants produced fewer callose-encased haustoria compared with wild type (Figure 5B). To determine if this difference was due to a reduced number of haustoria produced by *Hpa* on *pdlp1,2,3* mutants we co-stained infected tissue with trypan blue and aniline blue. Counts of aniline blue stained haustoria and trypan blue stained haustoria in a single image indicate that relative to Col-0, *pdlp1,2,3* mutants have a reduced proportion of encased haustoria (Figure S5). We also performed haustorial counts on the *pdlp1* mutant and double mutants *pdlp1,2*, *pdlp2,3* and *pdlp3,1*. None of these lines showed a significant difference in the proportion of encased haustoria relative to Col-0 (Figure S5), indicating that no single mutation present in these lines is responsible for the phenotype observed in the *pdlp1,2,3* mutants.

At higher magnification, the aniline blue stained encasement layer that surrounded haustoria in PDLP1 OE leaves appears thicker than that observed around haustoria in wild-type leaves (Figure 5C). A thin encasement is visible in *pdlp1,2,3* mutant leaves but they do not stain with aniline blue, suggesting a decrease or absence in callose accumulation around *Hpa* haustoria in the absence of PDLPs (Figure 5C). Thus, PDLPs are positive regulators of callose deposition during the encasement of *Hpa* haustoria.

**Figure 6:**
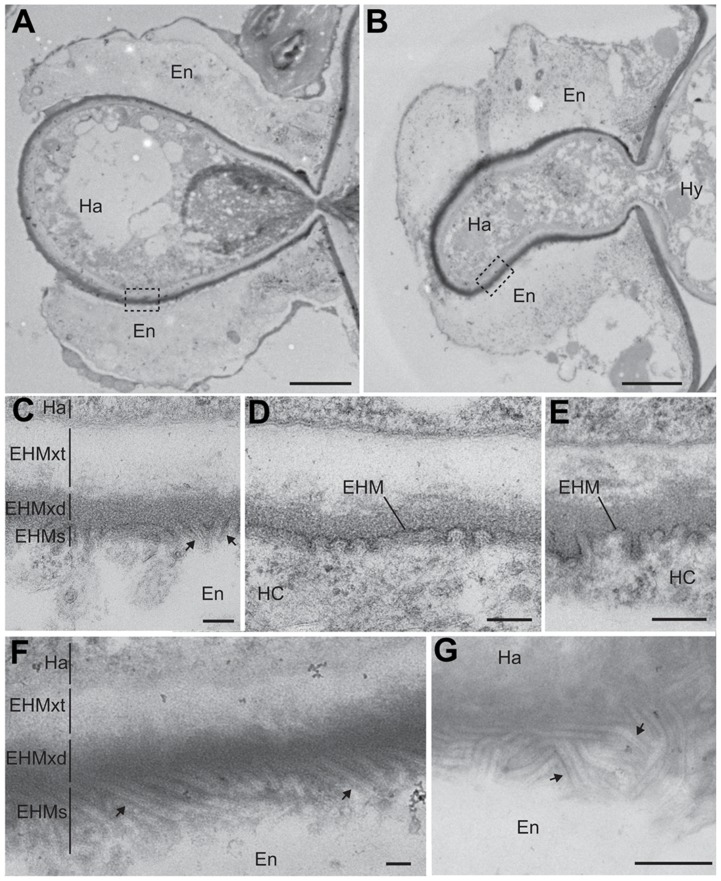
PDLP1 promotes membrane tubule formation at the extra-haustorial interface. Transmission electron micrographs of *Hpa* Waco9 haustoria observed in Col-0 (A) and PDLP1 OE (B) plants harvested 6 DPI. Boxes represent regions from which high magnification images (C, D, E, F) were taken. High magnification images of the host-pathogen interface in Col-0 (C–E) and PDLP1 OE (F) show that the EHMx and EHM forms an electron dense structure that has membrane invaginations (arrows) at the host surface. In regions in which the haustorium is encased the EHM is not continuously defined and may comprise the EHM and inner membrane of the encasement, thus this membrane is differentially denoted EHMs to allow for the possibility of multiple membrane layers. (F) Membrane invaginations are longer and more abundant in PDLP1 OE plants. (G) An oblique section of the surface of an haustorium in a PDLP1 OE cell illustrates the density and length of these protrusions. Ha, haustorium; En, encasement; EHMxt, extrahaustorial matrix translucent; EHMxd, extrahaustorial matrix dense. Scale bars are 2 µm (A and B), 100 nm (C–F) and 500 nm (E).

To further examine structural differences in encased haustoria in wild-type and PDLP1 OE plants, we next observed haustoria by transmission electron microscopy. In both encased and unencased haustoria, the EHMx appeared to consist of two layers that differ in electron density: an electron dense layer adjacent to the EHM and an electron translucent layer adjacent to the haustorial membrane (Figure 6). The translucent layer of the EHMx did not appear different in thickness or quality between wild-type and PDLP1 OE cells (Figure 6A–F) and may correspond with the haustorial wall [12]. However, while in wild-type plants the electron dense layer of the EHMx stained similarly to the plant cell wall, and may represent the true EHMx [12], this layer was frequently more densely stained relative to the host cell wall in PDLP1 OE plants (Figure 6B). At higher magnification, this increased staining density in the EHMx correlates with the presence of membrane invaginations at the boundary between the electron dense layer of the EHMx and the host cell (arrows, Figure 6C–G). When the haustorium is fully or partially encased, the model for haustorium formation would suggest that an additional membrane layer would be present here. In our images, each time the haustorium was encased (Figure 6C, F, G) we did not see clear evidence of an additional membrane layer but this may be due to the increased membrane convolution in these regions, or alternately poor membrane preservation. In the PDLP1 OE line, membrane invaginations are uniform in diameter (approximately 25 nm) and in an oblique section could be measured to be greater than 450 nm long (Figure 6G). Invaginations, or convolution of the EHM, were also observed in wild-type cells but when compared with haustoria in PDLP1 OE plants were less frequent and shorter in length (Figure 6C–E, Table S2).

Callose deposition is not always observed during infection of haustorium-forming pathogens. We tested whether PDLP1 plays a role during infection of *Albugo laibachii*, an Arabidopsis oomycete pathogen which forms haustoria in Arabidopsis mesophyll cells, but does not trigger callose deposition [57]. No PDLP1 signal at the EHM of *A. laibachii* haustoria could be observed (Figure S6) suggesting that PDLP1 localisation is specific to an *Hpa* response and/or callose deposition.

It has been established that during plant development, callose is deposited at PD where it regulates cell-to-cell communication [58], [59]. PDLP1 OE plants show reduced molecular flux *via* PD [33] and PDLP5 overexpression increases callose deposition at PD [36], so we asked whether PDLP1 also promotes callose deposition at PD. Qualitative assessment of aniline blue staining of PDLP1 OE plants showed that callose deposition was increased relative to wild-type plants but that this increase is not limited to PD – callose is deposited across the cell (Figure S7). mCherry-TMCT plants exhibited increased callose deposition by aniline blue staining but this callose appeared to be located in discrete membrane domains, likely PD (Figure S7). mCherry-TMCT plants also showed reduced intercellular flux via PD (Figure S7). Thus, as for PDLP5, it is likely that PDLP1 acts on the PD flux via callose deposition and that for PDLP1 this is mediated by the C-terminal domains of the protein.

## Discussion

Haustoria are the primary interface for molecular exchange between pathogen and host, for pathogen nutrient uptake [60]–[64], effector delivery [65]–[68] and targeted defence responses from the host [9], [69]. The encasement of haustoria by host cells has been observed in both compatible and incompatible interactions and can allow the host to suppress the growth of the pathogen [2], [9], [70], [71]. A variety of materials are deposited in haustorial encasements, including polysaccharides, proteins and membranous material, forming a barrier that is presumed to inhibit the loss of nutrients from the host and effector delivery from the pathogen. The beta-1,3-glucan callose is an abundant component of haustorial encasements but is not essential for their formation [72]. In this study we show that in the *Hpa*-Arabidopsis interaction, the *pdlp1,2,3* mutant has reduced callose content in encasements and increased susceptibility to *Hpa*. This is in contrast to the *pmr4* mutants, which similarly have reduced callose encasement of haustoria (Figure 5) but increased resistance to *Hpa* [55]. *pmr4* mutants exhibit enhanced SA-dependent defence responses [73] which offers an explanation for enhanced resistance in the absence of callose. The opposite effect on susceptibility evident in two mutants depleted in callose suggests that callose regulation of SA-triggered responses is dependent upon callose synthase, or non-haustorial callose. Further, the *pdlp1,2,3* mutant demonstrates that a callose-depleted encasement is less effective at impeding the pathogen and that callose is a critical component of targeted defence at haustoria in the *Hpa*-Arabidopsis interaction.

PDLPs were originally identified as a family of proteins that localise specifically at PD. They are a protein family of unknown function but have been associated with the regulation of molecular flux between cells via PD [33], virus tubule assembly at PD [34] and responses to both herbivores [38] and bacterial pathogens [36]. During *Hpa* infection, *PDLP1* expression is increased in infected cells, but expression of *PDLP2*, *PDLP3* or *PDLP5* is not. *PDLP5* expression is upregulated by SA [36]. It was recently demonstrated that *Hpa* effectors suppress the induction of a number of defence-responsive genes, including the SA responsive gene *PATHOGENESIS-RELATED GENE 1* [74], [75]. It is possible that effectors delivered from haustoria also block SA induction of *PDLP5* in infected cells.

We observed that while PDLP1-GFP is located at PD under normal conditions, upon infection with *Hpa* PDLP1-GFP is located at the EHM (Figure 2, 3 and S2). This association with the EHM was observed early in the infection, prior to haustorial encasement. PDLP1-GFP fluorescence was also associated with the encasement as it developed, and protein produced from native promoter expression could be resolved in two layers at the boundary of the encasement. No PDLP1-GFP could be seen at the haustorium in mature encasements (Figure 2). The EHM and PD are both specialised membrane domains that are continuous with the PM. Our data shows that while both membrane domains contain PDLPs, other proteins located in the plasmodesmal PM are not located in the EHM, indicating they differ in protein content. While no immediate similarity in the function of these membranes is apparent, this raises the possibility that PDLPs perform similar functions at PD and haustorial membranes.

While several contexts have been identified for PDLP function, we do not know the mode of activity for this family of proteins. Immunoprecipitation of PDLP1-GFP in infected and non-infected tissue did not identify any proteins that associate with PDLP1 specifically in infected tissue. This may be because cells harbouring haustoria are rare in comparison with the surrounding non-infected cells which might dilute the signal, or alternatively indicate that PDLP1 targeting in infected cells is a result of redirection of an endogenous pathway. VAMP721 was identified in PDLP1 containing membranes (Table 1) and has recently been found to be required for RPW8 targeting to the EHM of *G. orontii* haustoria. This allows the possibility that PDLP1 and RPW8 exploit the same trafficking pathway for delivery to the EHM, and that this pathway is required for defence during different host-pathogen interactions. VAMP721 is also required for cell plate formation, and in both samples PDLP1 immuno-purified with the cell-plate marker PATELLIN1. The related protein PATELLIN2 was also identified in the PD proteome [31], allowing the hypothesis that there is functional similarity between the membrane domains of haustoria, PD and the cell plate.

Callose deposition occurs at haustoria and PD, both membrane domains at which PDLP1 is observed. Here we have shown that PDLPs contribute to callose deposition in the encasement of *Hpa* haustoria. When we examined the callose content in haustorial encasements in the *pdlp1,2,3* mutant, we observed that they were thinner and contained less callose compared with wild-type plants (Figure 5), further confirming the correlation between PDLP activity and callose deposition. The specific role of PDLPs as relates to callose deposition is unclear. PDLP1 localisation at the EHM precedes callose deposition and then follows the encasement as it develops. The localisation of PDLP1-GFP at the EHM raises questions relating to the spatio-temporal role of PDLP1. It is clear that PDLP1 present in membranes of the developing encasement could directly regulate callose filling of the encasement. However, we saw no evidence of callose deposition at the EHM prior to encasement development and so the significance of this localisation remains undefined.

PDLP2 and PDLP3 (the genes for which are not significantly expressed in haustoria-containing cells), as well as the synthetic protein mCit-TMCT, also localise to the EHM suggesting that, as for PD targeting, haustorial targeting information is located within the transmembrane region and/or cytoplasmic tail of PDLPs. The observation that overexpression of the TMCT also increases PD associated callose and reduces intercellular flux indicates that the C-terminal domains of PDLPs are also sufficient to promote callose deposition.

Transmission electron microscopy of haustoria that form in PDLP1 overexpressors showed a proliferation of membrane as tubules or invaginations at the host interface. A convoluted or invaginated EHM has previously been observed in the *Hpa*-Arabidopsis interaction [15] as well as in the *Peronospora sp*-cabbage interaction [11], the *Albugo candida*-*Arabis alpina* interaction [57], the *G. orontii*-Arabidopsis interaction [24] and the *Puccinia coronata*-*Avena sativa* interaction [76] but to our knowledge, no molecular players involved in the genesis of these convolutions have been described so far. Our data suggest that overexpression of PDLP1 promotes the formation, stability and/or modification of the EHM such that a much greater surface area of host membrane is present around the haustoria. How this relates to callose filling of the encasement remains to be determined.

This study has identified that PDLP1 contributes to callose deposition at *Hpa* haustorial encasements and PDLPs are required for full defence against this pathogen. We have demonstrated that in the Arabidopsis-*Hpa* interaction callose deposition in the haustorial encasement is a key defence response and that PDLP function extends beyond the regulation of intercellular flux. It is not clear how PDLP activity regulates callose deposition but this study has identified the possibility that this process is common to different subcellular locations and stimuli.

## Materials and Methods

### DNA constructs and transgenic plants

*PDLP1* and *PDLP5* regulatory sequences were amplified from 1.5 kbp upstream of the ATG and cloned via Gateway Technology (Invitrogen) into the plant expression vector pKGWFS7 [77]. These constructs were used to generate stably expressing Arabidopsis by floral dipping [78]. The synthetic construct *SP-mCherry-TMCT*, which produces the protein mCherry-TMCT (mCh-TMCT) was made as described [33].

### Pathogen assays

*Hpa* isolates Noco2, Waco9 and Emoy2 were used in this study. For infection, 10 day old plants were spray-inoculated to saturation with a spore suspension of 5×10^4^ spores/ml. Plants were kept in a growth cabinet at 16°C for 3 to 6 days with a 10 h photoperiod. To evaluate conidiospore production, 10 pools of 2 plants were harvested in 1 mL of water for each line. After vortexing, the amount of liberated spores was determined with a haemocytometer as described by [79]. Statistical analyses have been performed in three independent experiments, using ANOVA. To evaluate conidiophore development, *Hpa* infection structures were stained by boiling for 2 min in lactophenol trypan blue (10% phenol, 10% glycerol, 0.01% trypan blue and 10% lactic acid). Samples were cleared in 15 M chloral hydrate and mounted in 60% glycerol. For *Hpa* Emoy2 infection in Col-0 and the transgenic lines, the number of conidiophores per cotyledons was scored by manually scanning the abaxial and adaxial surfaces of each cotyledon of 50 plants per transgenic line. Two biological replicates were performed. For the imaging of *Hpa* Emoy2 development in the cotyledons, the samples were observed with a Leica DM R microscope. Pictures were taken with a Leica DFC 300 FX Digital Camera.

### Histochemical localisation of GUS activity

GUS activity was assayed histochemically with 5-bromo-4-chloro-3-indolyl-β-d-glucuronic acid (1 mg/ml) in a buffer containing 100 mM sodium phosphate pH 7, 0.5 mM potassium ferrocyanide, 0.5 mM potassium ferricyanide, 10 mM EDTA, 0.1% Triton. *Arabidopsis* leaves were vacuum-infiltrated with staining solution and then incubated overnight at 37°C in the dark. Destaining was performed in 100% ethanol followed by incubation in chloral hydrate solution. Sections were observed with a Zeiss Axioplan 2 microscope (Jena, Germany).

### Callose staining and confocal microscopy

For callose staining of live infected tissue, 0.1% aniline blue [80] was pressure infiltrated into aerial tissues. For haustorial encasement quantification, infected leaves were stained with aniline blue as described [21] and stained encasements were quantified using CalloseMeasurer [56]. For *in vivo* localisation of fluorescent-tagged proteins in Arabidopsis, 10 day old infected seedlings were mounted in water and analysed on a Leica DM6000B/TCS SP5 (Leica Microsystems) or Zeiss LSM780 (Zeiss) confocal microscope. GFP was excited at 488 nm and collected at 515–525 nm; mCitrine and YFP were excited at 514 nm and collected at 525–540 nm; the aniline blue fluorochrome was excited with a 405 nm laser and collected at 440–490 nm.

### Transmission electron microscopy

Infected leaf samples were cut into 1 mm^3^ pieces and immediately placed in 2.5% (v/v) glutaraldehyde in 0.05 M sodium cacodylate, pH 7.3, with vacuum infiltration and then left overnight at room temperature to fix the tissue. Samples were rinsed in buffer, placed in 30% (v/v) ethanol on ice then transferred into flow-through capsules for further processing in a Leica AFS2 (Leica Microsystems) following a PLT protocol (progressive lowering of temperature) based on that described by [81]. This procedure was followed except for the following modifications; after dehydration through an ethanol series, infiltration steps were performed at −20°C with LR White resin plus 0.5% (w/v) benzoin methyl ether and polymerization was in Beem capsules, with indirect UV irradiation for 24 h at −20°C followed by 16 h at room temperature. The material was sectioned with a diamond knife using a Leica UC6 ultramicrotome (Leica Microsystems). Ultrathin sections of approximately 90 nm were picked up on 200 mesh copper grids which had been pyroxylin- and carbon-coated. The sections were stained with 2% (w/v) uranyl acetate for 1 h and 1% (w/v), lead citrate for 1 min and then washed in water and air dried. Grids were viewed in a FEI Tecnai 20 transmission electron microscope (FEI) at 200 kV and imaged using an AMT XR60 digital camera (Deben) to record TIF files.

### Membrane purification and co-immunoprecipitation

For co-immunoprecipitation, 3–6 g of leaf material was ground to a fine powder in liquid nitrogen and resuspended in extraction buffer [50 mM Tris-HCl pH 7.5, 150 mM NaCl, 10% glycerol, 1 mM EDTA, 5 mM DTT, 1% IGEPAL CA630 (Sigma), 1× protease inhibitor cocktail for plant cell extracts (Sigma)] at a ratio of 2 mL buffer/1 g leaf tissue. The homogenized extract was centrifuged at 4°C/20000×*g/*20 min and the supernatant was passed through a double layer of Miracloth. GFP-tagged proteins were immunoprecipitated by adding 25 µL of GFP-Trap beads (Chromotek) followed by incubation on a rolling wheel at 4°C for 2 h. The beads were collected by centrifugation at 4°C/1000×*g/*2 min, resuspended in 1 mL extraction buffer and transferred to 1.5 mL tubes. The beads were washed by 3 further rounds of centrifugation (4°C/1000×*g/*1 min) followed by resuspension in 1 ml extraction buffer. To extract proteins, the beads were boiled for 5 min in 45 µL of 1× SDS sample buffer.

### Mass spectrometry

Samples for LC MS analysis were prepared by excising bands from one dimensional SDS-PAGE gels stained with colloidal Coomassie Brilliant Blue (Simply Blue Safe stain, Invitrogen). The gel slices were destained in 50% acetonitrile, and cysteine residues modified by 30 min reduction in 10 mM DTT followed by 20 min alkylation with 55 mM choroacetamide. After extensive washing with destaining solvent and 100% acetonintrile, gel pieces were incubated with trypsin (Promega) in 100 mM ammonium bicarbonate and 5% acetonitrile in water at 37°C overnight. Generated peptides were extracted with solution of 5% formic acid and 50% acetonitrile, evaporated to dryness, and dissolved in 2% acetonitrile, 0.1% trifluoroacetic acid prior LC-MS/MS analysis.

LC-MS/MS analysis was performed using a hybrid mass spectrometer LTQ-Orbitrap XL (ThermoFisher Scientific) and a nanoflow UHPLC system (nanoAcquity, Waters Corp.) The peptides were applied to a reverse phase trap column (Symmetry C18, 5 µm, 180 µm ×20 mm, Waters Corp.) connected to an analytical column (BEH 130 C18, 1.7 µm, 75 µm ×250 mm, Waters Corp.) in vented configuration using nano-T coupling union. Peptides were eluted in a gradient of 3–40% acetonitrile in 0.1% formic (solvent B) acid over 50 min followed by a gradient of 40–60% B over 3 min at a flow rate of 250 nL min^−1^ at 40°C. The mass spectrometer was operated in positive ion mode with nano-electrospray ion source with ID 20 µm fused silica emitter (New Objective). Voltage +2 kV was applied via platinum wire held in PEEK T-shaped coupling union. Transfer capillary temperature was set to 200°C, no sheath gas, and the focusing voltages in factory default setting were used. In the Orbitrap, MS scan resolution of 60,000 at 400 m/z, range m/z 300 to 2000, automatic gain control (AGC) target 1000000 counts, and maximum inject time to 1000 ms were set. In the linear ion trap (LTQ) the normal scan rate and normal range, AGC accumulation target 30,000 counts, and maximum inject time to 150 ms were used. A data dependent algorithm was used to trigger and measure up to 5 tandem MS spectra in the ion trap from all precursor ions detected in master scan in the Orbitrap. Following function and detailed settings were used: Orbitrap pre-scan function, isolation width 2 *m/z* and collision energy set to 35%, and precursor ion collision threshold 1000 counts. The selected ions were fragmented in the ion trap using collision induced dissociation (CID). Dynamic exclusion was enabled allowing for 1 repeat, with a 60 sec exclusion time, and maximal size of dynamic exclusion list 500 items. Chromatography function to trigger MS/MS event close to the peak summit was used with correlation set to 0.9, and expected peak width 7 s. Charge state screening enabled allowed only higher than 2+ charge states to be selected for MS/MS fragmentation.

### Software processing and peptide identification

Peak lists in format of Mascot generic files (.mgf files) were prepared from raw data using Proteome Discoverer v1.2 (ThermoFisher Scientific) and concatenated using in house developed Perl script. Peak picking settings were as follows: range m/z 300–5000, minimum number of peaks in a spectrum was set to 1, S/N threshold for Orbitrap spectra set to 1.5, and automatic treatment of unrecognized charge states was used. Peak lists were searched on Mascot server v.2.4.1 (Matrix Science) against TAIR (version 10) database with added constructs that were used throughout the experiments. Tryptic peptides only, up to 2 possible miscleavages and charge states +2, +3, +4 were allowed in the search. The following modifications were included in the search: oxidized methionine (variable), carbamidomethylated cysteine (static). Data were searched with a monoisotopic precursor and fragment ions mass tolerance 10 ppm and 0.8 Da respectively. Mascot results were combined in Scaffold v. 4 (Proteome Software, [82]) and exported in Excel (Microsoft Office). Peptide identifications were accepted if they could be established at greater than 95.0% probability by the Peptide Prophet algorithm [83] with Scaffold delta-mass correction. Protein identifications were accepted if they could be established at greater than 99.0% probability and contained at least 2 identified unique peptides. Protein probabilities were assigned by the Protein Prophet algorithm [84]. Proteins that contained similar peptides and could not be differentiated based on MS/MS analysis alone were grouped to satisfy the principles of parsimony.

## Supporting Information

Figure S1 **Changes in gene expression of** ***PDLP*** **genes 3 and 5 days post inoculation (DPI) with** ***Hpa*** **Waco9.** Transcript levels (log2 value) [39] are represented by a heat map where transcript levels relative to mock treated samples are represented in red to indicate increased expression and green to indicate decreased expression.
(TIF)

Figure S2 **Haustorial association of PDLP1-GFP when expressed under the native promoter.** (A) PDLP1-GFP (green) is present surrounding the haustorium before and during encasement. Haustoria (indicated as I, II and III in the brightfield image in the top panel) in varying stages of encasement each (aniline blue, blue) show PDLP1-GFP fluorescence surrounding the haustorium. Haustoria I–III show increasing levels of encasement. The bottom panel shows the overlay of PDLP1-GFP and aniline blue, red is chlorophyll autofluorescence. (B) In developing encasements PDLP1-GFP fluorescence can be resolved into two layers which are presumably membrane layers surrounding the encasement. Scale bars are 10 µm.
(TIF)

Figure S3 **Subcellular localisation of the markers used in this study in non-infected cells.** PDLP2-GFP, PDLP3-GFP, mCit-TMCT, PDCB1-mCit and MP17-GFP are all located at PD. TET3-YFP is visible in the PM and PD. PD are indicated by arrows, scale bars are 10 µm.
(TIF)

Figure S4 **PDLPs positively regulate immunity to** ***Hpa*** **Emoy2.** Conidiophore counting on cotyledons 6 DPI with *Hpa* Emoy2 on Col-0 and *pdlp1,2,3* mutant plants. Error bars are the standard error of the mean, * indicates p-value <0.05 by non-parametric Mann-Whitney analysis.
(TIF)

Figure S5 **Reduced callose encasement in** ***pdlp1,2,3*** **mutants is not due to reduced number of haustoria.** (A) Number of haustoria (*Hpa* Noco) stained with aniline blue (encased haustoria) relative to the number of haustoria stained with trypan blue (total haustoria) for Col-0, the single knockout mutant *pdlp1*, the double knockout mutants *pdlp1,2*, *pdlp2,3*, *pdlp3,1* and the triple knockout mutant *pdlp1,2,3*. Only the triple knockout *pdlp1,2,3* shows a reduced proportion of encased haustoria. (B) The total number of haustoria per image for each genotype. *pdlp1,2,3* mutant plants showed a greater number of haustoria per image, * indicates p-value <0.05 and *** indicates p-value <0.001 by Student's t-test.
(TIF)

Figure S6 **PDLP1-GFP does not localise to the EHM of** ***Albugo laibachii*** **haustoria.** Some PDLP1-GFP fluorescence (left) is located at the neck of the haustorium but no label is seen around the periphery of the haustorium. The transmitted light image overlayed with the fluorescence image (right) identifies the position of the haustorium (asterisk). Scale bar is 5 µm.
(TIF)

Figure S7 **Overexpression of the PDLP1 TM and CT increases callose deposition at PD and reduces cell-to-cell flux.** (A) Aniline blue staining of leaves of Col-0, PDLP1 OE and mCherry (mCh)-TMCT identifies that callose deposition is increased in both PDLP1 OE and mCh-TMCT plants relative to Col-0. In PDLP1 OE this staining is evident around the whole cell while in the mCh-TMCT leaves staining is discretely located at PD. Scale bars are 20 µm. (B) Microprojectile bombardment assays indicate that GFP diffusion from a bombardment site is reduced in mCh-TMCT leaves relative to Col-0.
(TIF)

Table S1 **Peptides identified by MS/MS.** Total spectrum counts and mascot ion score for each peptide detected in control and experimental samples.
(XLSX)

Table S2 **Frequency and length of membrane invaginations observed by transmission electron microscopy.** The total number of haustoria imaged by transmission electron microscopy for Col-0 and PDLPOE plants, and the frequency of membrane invaginations less than 100 nm and greater than 100 nm observed in these samples.
(DOCX)

Methods S1 **Materials and methods used to generate data contained in the Supporting Information file.**
(DOCX)

## References

[1] Hahn M, Mendgen K (2001) Signal and nutrient exchange at biotrophic plant-fungus interfaces. Curr Opin Plant Biol 4: 322–327. 11418342 10.1016/s1369-5266(00)00180-1

[2] Meyer D, Pajonk S, Micali C, O'Connell R, Schulze-Lefert P (2009) Extracellular transport and integration of plant secretory proteins into pathogen-induced cell wall compartments. Plant J 57: 986–999. 19000165 10.1111/j.1365-313X.2008.03743.x

[3] Caillaud MC, Piquerez SJ, Fabro G, Steinbrenner J, Ishaque N, et al (2012) Subcellular localization of the Hpa RxLR effector repertoire identifies a tonoplast-associated protein HaRxL17 that confers enhanced plant susceptibility. Plant J 69: 252–265. 21914011 10.1111/j.1365-313X.2011.04787.x

[4] Lu YJ, Schornack S, Spallek T, Geldner N, Chory J, et al (2012) Patterns of plant subcellular responses to successful oomycete infections reveal differences in host cell reprogramming and endocytic trafficking. Cell Microbiol 14: 682–697. 22233428 10.1111/j.1462-5822.2012.01751.x PMC4854193

[5] Harder DE, Chong J (1991) Rust Haustoria. In: Mendgen K, Lesemann D-E, editors. Electron microscopy of plant pathogens: Springer Berlin Heidelberg. pp.235–250.

[6] Knauf GM, Welter K, Müller M, Mendgen K (1989) The haustorial host-parasite interface in rust-infected bean leaves after high-pressure freezing. Physiol Mol Plant Pathol 34: 519–530.

[7] Perfect SE, Green JR (2001) Infection structures of biotrophic and hemibiotrophic fungal plant pathogens. Mol Plant Pathol 2: 101–108. 20572997 10.1046/j.1364-3703.2001.00055.x

[8] Koh S, Andre A, Edwards H, Ehrhardt D, Somerville S (2005) *Arabidopsis thaliana* subcellular responses to compatible *Erysiphe cichoracearum* infections. Plant J 44: 516–529. 16236160 10.1111/j.1365-313X.2005.02545.x

[9] Wang W, Wen Y, Berkey R, Xiao S (2009) Specific targeting of the Arabidopsis resistance protein RPW8.2 to the interfacial membrane encasing the fungal haustorium renders broad-spectrum resistance to powdery mildew. Plant Cell 21: 2898–2913. 19749153 10.1105/tpc.109.067587 PMC2768920

[10] Roberts AM, Mackie AJ, Hathaway V, Callow JA, Green JR (1993) Molecular differentiation in the extrahaustorial membrane of pea powdery mildew haustoria at early and late stages of development. Physiol Mol Plant Pathol 43: 147–160.

[11] Chou CK (1970) An electron-microscope study of host penetration and early stages of haustorium formation of *Peronospora parasitica* (Fr.) tul. on cabbage cotyledons. Ann Bot 34: 189–204.

[12] Mims CW, Rodriguez-Lother C, Richardson EA (2002) Ultrastructure of the host-pathogen interface in daylily leaves infected by the rust fungus *Puccinia hemerocallidis* . Protoplasma 219: 221–226. 12099222 10.1007/s007090200023

[13] Heath MC (1976) Ultrastructural and functional similarity of the haustorial neckband of rust fungi and the Casparian strip of vascular plants. Can J Bot 54: 2484–2489.

[14] Harder D E, J C (1984) Structure and physiology of haustoria. Bushnell W. R, Roelfs A. P, editors. Orlando, FL, USA: Academic Press, Inc, pp.431–476.

[15] Mims CW, Richardson EA, Holt BF 3rd, Dangl JL (2004) Ultrastructure of the host–pathogen interface in *Arabidopsis thaliana* leaves infected by the downy mildew *Hyaloperonospora parasitica* . Can J Bot 82: 1001–1008.

[16] Luna E, Pastor V, Robert J, Flors V, Mauch-Mani B, et al (2011) Callose deposition: a multifaceted plant defense response. Mol Plant Microbe Interact 24: 183–193. 20955078 10.1094/MPMI-07-10-0149

[17] Eulgem T, Rushton PJ, Schmelzer E, Hahlbrock K, Somssich IE (1999) Early nuclear events in plant defence signalling: rapid gene activation by WRKY transcription factors. EMBO J 18: 4689–4699. 10469648 10.1093/emboj/18.17.4689 PMC1171542

[18] Ton J, Flors V, Mauch-Mani B (2009) The multifaceted role of ABA in disease resistance. Trends Plant Sci 14: 310–317. 19443266 10.1016/j.tplants.2009.03.006

[19] Torres MA, Jones JD, Dangl JL (2006) Reactive oxygen species signaling in response to pathogens. Plant Physiol 141: 373–378. 16760490 10.1104/pp.106.079467 PMC1475467

[20] Dong X, Hong Z, Chatterjee J, Kim S, Verma DP (2008) Expression of callose synthase genes and its connection with Npr1 signaling pathway during pathogen infection. Planta 229: 87–98. 18807070 10.1007/s00425-008-0812-3

[21] Caillaud MC, Piquerez SJ, Jones JD (2012) Characterization of the membrane-associated HaRxL17 Hpa effector candidate. Plant Signal Behav 7: 145–149. 22301983 10.4161/psb.7.1.18450 PMC3357357

[22] Fellbrich G, Romanski A, Varet A, Blume B, Brunner F, et al (2002) NPP1, a Phytophthora-associated trigger of plant defense in parsley and Arabidopsis. Plant Journal 32: 375–390. 12410815 10.1046/j.1365-313x.2002.01454.x

[23] Soylu EM, Soylu S (2003) Light and electron microscopy of the compatible interaction between Arabidopsis and the downy mildew pathogen *Peronospora parasitica* . J Phytopathol-Phytopathologische Zeitschrift 151: 300–306.

[24] Micali CO, Neumann U, Grunewald D, Panstruga R, O'Connell R (2011) Biogenesis of a specialized plant-fungal interface during host cell internalization of *Golovinomyces orontii* haustoria. Cell Microbiol 13: 210–226. 20880355 10.1111/j.1462-5822.2010.01530.x

[25] Donofrio NM, Delaney TP (2001) Abnormal callose response phenotype and hypersusceptibility to *Peronospora parasitica* in defense-compromised Arabidopsis *nim1-1* and salicylate hydroxylase-expressing plants. Molecular Plant-Microbe Interactions 14: 439–450. 11310731 10.1094/MPMI.2001.14.4.439

[26] Keinath NF, Kierszniowska S, Lorek J, Bourdais G, Kessler SA, et al (2010) PAMP (pathogen-associated molecular pattern)-induced changes in plasma membrane compartmentalization reveal novel components of plant immunity. J Biol Chem 285: 39140–39149. 20843791 10.1074/jbc.M110.160531 PMC2998143

[27] Stahl Y, Grabowski S, Bleckmann A, Kuhnemuth R, Weidtkamp-Peters S, et al (2013) Moderation of Arabidopsis root stemness by CLAVATA1 and ARABIDOPSIS CRINKLY4 receptor kinase complexes. Curr Biol 23: 362–371. 23394827 10.1016/j.cub.2013.01.045

[28] Tilsner J, Amari K, Torrance L (2011) Plasmodesmata viewed as specialised membrane adhesion sites. Protoplasma 248: 39–60. 20938697 10.1007/s00709-010-0217-6

[29] Faulkner C (2013) Receptor-mediated signaling at plasmodesmata. Front Plant Sci 4: 521. 24385979 10.3389/fpls.2013.00521 PMC3866517

[30] Raffaele S, Bayer E, Lafarge D, Cluzet S, German Retana S, et al (2009) Remorin, a solanaceae protein resident in membrane rafts and plasmodesmata, impairs potato virus X movement. Plant Cell 21: 1541–1555. 19470590 10.1105/tpc.108.064279 PMC2700541

[31] Fernandez-Calvino L, Faulkner C, Walshaw J, Saalbach G, Bayer E, et al (2011) Arabidopsis plasmodesmal proteome. PLoS One 6: e18880. 21533090 10.1371/journal.pone.0018880 PMC3080382

[32] Maule A, Faulkner C, Benitez-Alfonso Y (2012) Plasmodesmata "in Communicado". Front Plant Sci 3: 30. 22645579 10.3389/fpls.2012.00030 PMC3355775

[33] Thomas CL, Bayer EM, Ritzenthaler C, Fernandez-Calvino L, Maule AJ (2008) Specific targeting of a plasmodesmal protein affecting cell-to-cell communication. PLoS Biol 6: e7. 18215111 10.1371/journal.pbio.0060007 PMC2211546

[34] Amari K, Boutant E, Hofmann C, Schmitt-Keichinger C, Fernandez-Calvino L, et al (2010) A family of plasmodesmal proteins with receptor-like properties for plant viral movement proteins. PLoS Pathog 6: e1001119. 20886105 10.1371/journal.ppat.1001119 PMC2944810

[35] Bayer E, Thomas C, Maule A (2008) Symplastic domains in the Arabidopsis shoot apical meristem correlate with PDLP1 expression patterns. Plant Signal Behav 3: 853–855. 19704520 10.4161/psb.3.10.6020 PMC2634395

[36] Lee JY, Wang X, Cui W, Sager R, Modla S, et al (2011) A plasmodesmata-localized protein mediates crosstalk between cell-to-cell communication and innate immunity in Arabidopsis. Plant Cell 23: 3353–3373. 21934146 10.1105/tpc.111.087742 PMC3203451

[37] Zavaliev R, Ueki S, Epel BL, Citovsky V (2011) Biology of callose (beta-1,3-glucan) turnover at plasmodesmata. Protoplasma 248: 117–130. 21116665 10.1007/s00709-010-0247-0 PMC9473521

[38] Bricchi I, Occhipinti A, Bertea CM, Zebelo SA, Brillada C, et al (2013) Separation of early and late responses to herbivory in Arabidopsis by changing plasmodesmal function. Plant J 73: 14–25. 22775399 10.1111/j.1365-313X.2012.05103.x

[39] Asai S, Piquerez SJM, Rallapallia G, Caillaud MC, Furzer O, et al (2014) Expression profiling during Arabidopsis/downy mildew interaction uncovers a highly-expressed effector which reduces salicylic acid-triggered immunity. PLoS Pathogens 10: e1004443 DOI:10.1371/journal.ppat.1004443 25329884 10.1371/journal.ppat.1004443 PMC4199768

[40] Vogel F, Hofius D, Sonnewald U (2007) Intracellular trafficking of potato leafroll virus movement protein in transgenic Arabidopsis. Traffic 8: 1205–1214. 17631001 10.1111/j.1600-0854.2007.00608.x

[41] Simpson C, Thomas C, Findlay K, Bayer E, Maule AJ (2009) An Arabidopsis GPI-anchor plasmodesmal neck protein with callose binding activity and potential to regulate cell-to-cell trafficking. Plant Cell 21: 581–594. 19223515 10.1105/tpc.108.060145 PMC2660613

[42] van der Biezen EA, Freddie CT, Kahn K, Parker JE, Jones JD (2002) Arabidopsis RPP4 is a member of the RPP5 multigene family of TIR-NB-LRR genes and confers downy mildew resistance through multiple signalling components. Plant J 29: 439–451. 11846877 10.1046/j.0960-7412.2001.01229.x

[43] Mukhtar MS, Carvunis AR, Dreze M, Epple P, Steinbrenner J, et al (2011) Independently evolved virulence effectors converge onto hubs in a plant immune system network. Science 333: 596–601. 21798943 10.1126/science.1203659 PMC3170753

[44] Stein M, Dittgen J, Sanchez-Rodriguez C, Hou BH, Molina A, et al (2006) Arabidopsis PEN3/PDR8, an ATP binding cassette transporter, contributes to nonhost resistance to inappropriate pathogens that enter by direct penetration. Plant Cell 18: 731–746. 16473969 10.1105/tpc.105.038372 PMC1383646

[45] Collins NC, Thordal-Christensen H, Lipka V, Bau S, Kombrink E, et al (2003) SNARE-protein-mediated disease resistance at the plant cell wall. Nature 425: 973–977. 14586469 10.1038/nature02076

[46] Kohorn BD, Kohorn SL, Todorova T, Baptiste G, Stansky K, et al (2012) A dominant allele of Arabidopsis pectin-binding wall-associated kinase induces a stress response suppressed by MPK6 but not MPK3 mutations. Mol Plant 5: 841–851. 22155845 10.1093/mp/ssr096 PMC3399699

[47] Liu J, Elmore JM, Fuglsang AT, Palmgren MG, Staskawicz BJ, et al (2009) RIN4 functions with plasma membrane H+-ATPases to regulate stomatal apertures during pathogen attack. PLoS Biol 7: e1000139. 19564897 10.1371/journal.pbio.1000139 PMC2694982

[48] Kim H, O'Connell R, Maekawa-Yoshikawa M, Uemura T, Neumann U, et al (2014) The powdery mildew resistance protein RPW8.2 is carried on VAMP721/722 vesicles to the extrahaustorial membrane of haustorial complexes. Plant J 79: 835–847 DOI: 10.1111/tpj.12591 24941879 10.1111/tpj.12591

[49] Kwon C, Neu C, Pajonk S, Yun HS, Lipka U, et al (2008) Co-option of a default secretory pathway for plant immune responses. Nature 451: 835–840. 18273019 10.1038/nature06545

[50] Nagasaki N, Tomioka R, Maeshima M (2008) A hydrophilic cation-binding protein of *Arabidopsis thaliana*, AtPCaP1, is localized to plasma membrane via N-myristoylation and interacts with calmodulin and the phosphatidylinositol phosphates PtdIns(3,4,5)P(3) and PtdIns(3,5)P(2). FEBS J 275: 2267–2282. 18397324 10.1111/j.1742-4658.2008.06379.x

[51] Carman GM, Han GS (2006) Roles of phosphatidate phosphatase enzymes in lipid metabolism. Trends Biochem Sci 31: 694–699. 17079146 10.1016/j.tibs.2006.10.003 PMC1769311

[52] Peterman TK, Ohol YM, McReynolds LJ, Luna EJ (2004) Patellin1, a novel Sec14-like protein, localizes to the cell plate and binds phosphoinositides. Plant Physiol 136: 3080–3094 discussion 3001–3082. 15466235 10.1104/pp.104.045369 PMC523369

[53] El Kasmi F, Krause C, Hiller U, Stierhof YD, Mayer U, et al (2013) SNARE complexes of different composition jointly mediate membrane fusion in Arabidopsis cytokinesis. Mol Biol Cell 24: 1593–1601. 23515225 10.1091/mbc.E13-02-0074 PMC3655819

[54] Zhang L, Zhang H, Liu P, Hao H, Jin JB, et al (2011) Arabidopsis R-SNARE proteins VAMP721 and VAMP722 are required for cell plate formation. PLoS One 6: e26129. 22022536 10.1371/journal.pone.0026129 PMC3191180

[55] Vogel J, Somerville S (2000) Isolation and characterization of powdery mildew-resistant Arabidopsis mutants. Proc Natl Acad Sci U S A 97: 1897–1902. 10677553 10.1073/pnas.030531997 PMC26533

[56] Zhou J, Spallek T, Faulkner C, Robatzek S (2012) CalloseMeasurer: a novel software solution to measure callose deposition and recognise spreading callose patterns. Plant Methods 8: 49. 23244621 10.1186/1746-4811-8-49 PMC3571893

[57] Baka ZA (2008) Occurrence and ultrastructure of *Albugo candida* on a new host, *Arabis alpina* in Saudi Arabia. Micron 39: 1138–1144. 18640046 10.1016/j.micron.2008.06.001

[58] Benitez-Alfonso Y, Faulkner C, Pendle A, Miyashima S, Helariutta Y, et al (2013) Symplastic intercellular connectivity regulates lateral root patterning. Dev Cell 26: 136–147. 23850190 10.1016/j.devcel.2013.06.010

[59] Guseman JM, Lee JS, Bogenschutz NL, Peterson KM, Virata RE, et al (2010) Dysregulation of cell-to-cell connectivity and stomatal patterning by loss-of-function mutation in Arabidopsis *chorus* (*glucan synthase-like 8*). Development 137: 1731–1741. 20430748 10.1242/dev.049197

[60] Hahn M, Neef U, Struck C, Gottfert M, Mendgen K (1997) A putative amino acid transporter is specifically expressed in haustoria of the rust fungus *Uromyces fabae* . Mol Plant-Microbe Interact 10: 438–445. 9150593 10.1094/MPMI.1997.10.4.438

[61] Voegele RT, Struck C, Hahn M, Mendgen K (2001) The role of haustoria in sugar supply during infection of broad bean by the rust fungus *Uromyces fabae* . Proc Natl Acad Sci USA 98: 8133–8138. 11390980 10.1073/pnas.131186798 PMC35480

[62] Voegele RT, Wirsel S, Moll U, Lechner M, Mendgen K (2006) Cloning and characterization of a novel invertase from the obligate biotroph Uromyces fabae and analysis of expression patterns of host and pathogen invertases in the course of infection. Mol Plant-Microbe Interact 19: 625–634. 16776296 10.1094/MPMI-19-0625

[63] Struck C, Hahn M, Mendgen K (1996) Plasma membrane H+-ATPase activity in spores, germ tubes, and haustoria of the rust fungus Uromyces viciae-fabae. Fungal Gen Biol 20: 30–35. 10.1006/fgbi.1996.0006 8812284

[64] Struck C, Siebels C, Rommel O, Wernitz M, Hahn M (1998) The plasma membrane H+-ATPase from the biotrophic rust fungus *Uromyces fabae*: Molecular characterization of the gene (PMA1) and functional expression of the enzyme in yeast. Mol Plant-Microbe Interact 11: 458–465. 9612944 10.1094/MPMI.1998.11.6.458

[65] Catanzariti AM, Dodds PN, Ellis JG (2007) Avirulence proteins from haustoria-forming pathogens. FEMS Microbiol Lett 269: 181–188. 17343675 10.1111/j.1574-6968.2007.00684.x

[66] Dodds PN, Rafiqi M, Gan PH, Hardham AR, Jones DA, et al (2009) Effectors of biotrophic fungi and oomycetes: pathogenicity factors and triggers of host resistance. New Phytol 183: 993–1000. 19558422 10.1111/j.1469-8137.2009.02922.x

[67] Link TI, Voegele RT (2008) Secreted proteins of Uromyces fabae: similarities and stage specificity. Mol Plant Pathol 9: 59–66. 18705884 10.1111/j.1364-3703.2007.00448.x PMC6640452

[68] Whisson SC, Boevink PC, Moleleki L, Avrova AO, Morales JG, et al (2007) A translocation signal for delivery of oomycete effector proteins into host plant cells. Nature 450: 115–118. 17914356 10.1038/nature06203

[69] Bozkurt TO, Schornack S, Win J, Shindo T, Ilyas M, et al (2011) *Phytophthora infestans* effector AVRblb2 prevents secretion of a plant immune protease at the haustorial interface. Proc Natl Acad Sci U S A 108: 20832–20837. 22143776 10.1073/pnas.1112708109 PMC3251060

[70] Zhang H, Wang C, Cheng Y, Chen X, Han Q, et al (2012) Histological and cytological characterization of adult plant resistance to wheat stripe rust. Plant Cell Rep 31: 2121–2137. 22833277 10.1007/s00299-012-1322-0

[71] Zhang H, Wang C, Cheng Y, Wang X, Li F, et al (2011) Histological and molecular studies of the non-host interaction between wheat and *Uromyces fabae* . Planta 234: 979–991. 21691848 10.1007/s00425-011-1453-5

[72] Jacobs AK, Lipka V, Burton RA, Panstruga R, Strizhov N, et al (2003) An Arabidopsis callose synthase, GSL5, is required for wound and papillary callose formation. Plant Cell 15: 2503–2513. 14555698 10.1105/tpc.016097 PMC280557

[73] Nishimura MT, Stein M, Hou BH, Vogel JP, Edwards H, et al (2003) Loss of a callose synthase results in salicylic acid-dependent disease resistance. Science 301: 969–972. 12920300 10.1126/science.1086716

[74] Anderson RG, Casady MS, Fee RA, Vaughan MM, Deb D, et al (2012) Homologous RXLR effectors from *Hyaloperonospora arabidopsidis* and *Phytophthora sojae* suppress immunity in distantly related plants. Plant J 72: 882–893. 22709376 10.1111/j.1365-313X.2012.05079.x

[75] Caillaud MC, Asai S, Rallapalli G, Piquerez S, Fabro G, et al (2013) A downy mildew effector attenuates salicylic acid-triggered immunity in Arabidopsis by interacting with the host mediator complex. PLoS Biol 11: e1001732. 24339748 10.1371/journal.pbio.1001732 PMC3858237

[76] Chong J, Harder (1982) Ultrastructure of haustorium development in *Puccinia coronata* avenae: some host responses. Phytopathology: 1527–1533.

[77] Karimi M, Inze D, Depicker A (2002) GATEWAY vectors for Agrobacterium-mediated plant transformation. Trends Plant Sci 7: 193–195. 11992820 10.1016/s1360-1385(02)02251-3

[78] Clough SJ, Bent AF (1998) Floral dip: a simplified method for Agrobacterium-mediated transformation of *Arabidopsis thaliana* . Plant J 16: 735–743. 10069079 10.1046/j.1365-313x.1998.00343.x

[79] Robert-Seilaniantz A, Maclean D, Jikumaru Y, Hill L, Yamaguchi S, et al (2011) The microRNA miR393 redirects secondary metabolite biosynthesis away from camalexin and towards glucosinolates. Plant J 67: 218–31. 21457368 10.1111/j.1365-313X.2011.04591.x

[80] Thistlethwaite P, Porter I, Evans N (1986) Photophysics of the aniline blue fluorophore - a fluorescent-probe showing specificity toward (1-]3)-beta-D-glucans. J Phys Chem 90: 5058–5063.

[81] Wells B (1985) Low temperature box and tissue handling device for embedding biological tissue for immunostaining in electron microscopy. Micron and Microscopica Acta 16: 49–53.

[82] Searle BC (2010) Scaffold: a bioinformatic tool for validating MS/MS-based proteomic studies. Proteomics 10: 1265–1269. 20077414 10.1002/pmic.200900437

[83] Keller A, Nesvizhskii AI, Kolker E, Aebersold R (2002) Empirical statistical model to estimate the accuracy of peptide identifications made by MS/MS and database search. Anal Chem 74: 5383–5392. 12403597 10.1021/ac025747h

[84] Nesvizhskii AI, Keller A, Kolker E, Aebersold R (2003) A statistical model for identifying proteins by tandem mass spectrometry. Anal Chem 75: 4646–4658. 14632076 10.1021/ac0341261

