# Supplementary figures and images for: The Plasmodesmal Protein PDLP1 Localises to Haustoria-Associated Membranes during Downy Mildew Infection and Regulates Callose Deposition
Source: PLoS Pathog. 2014 Nov 13;10(11):e1004496. doi: 10.1371/journal.ppat.1004496 (PMC4231120; doi:10.1371/journal.ppat.1004496)

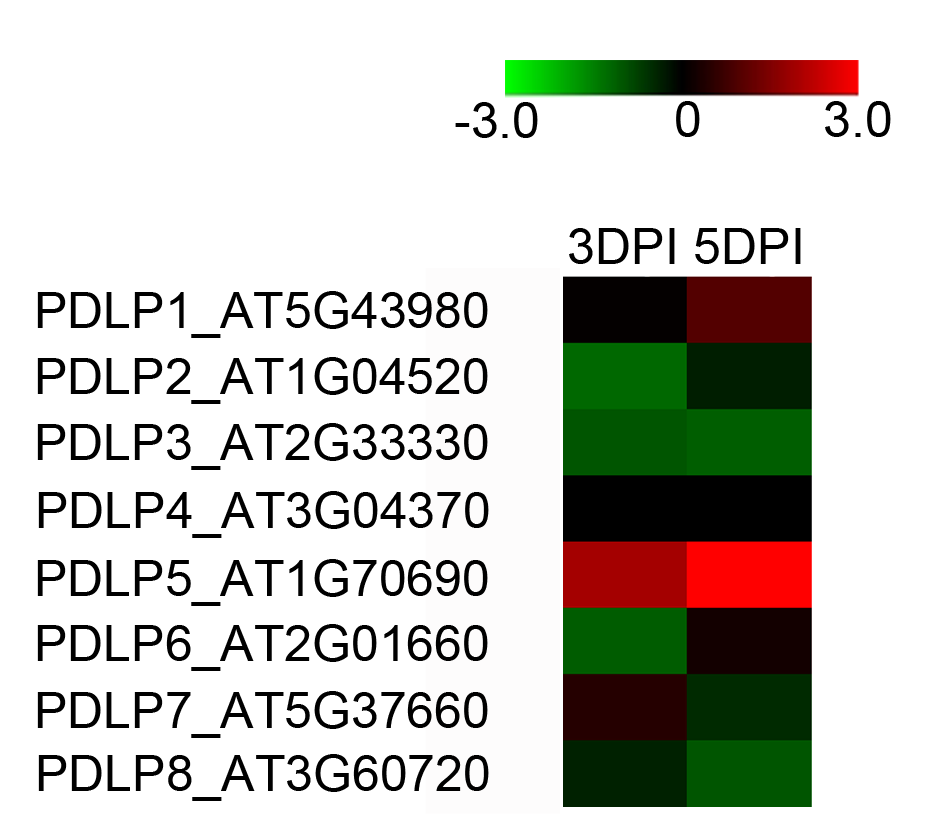

Supplement: Figure S1 — Changes in gene expression of PDLP genes 3 and 5 days post inoculation (DPI) with Hpa Waco9. Transcript levels (log2 value) [39] are represented by a heat map where transcript levels relative to mock treated samples are represented in red to indicate increased expression and green to indicate decreased expression. (TIF) [file ppat.1004496.s001.tif]

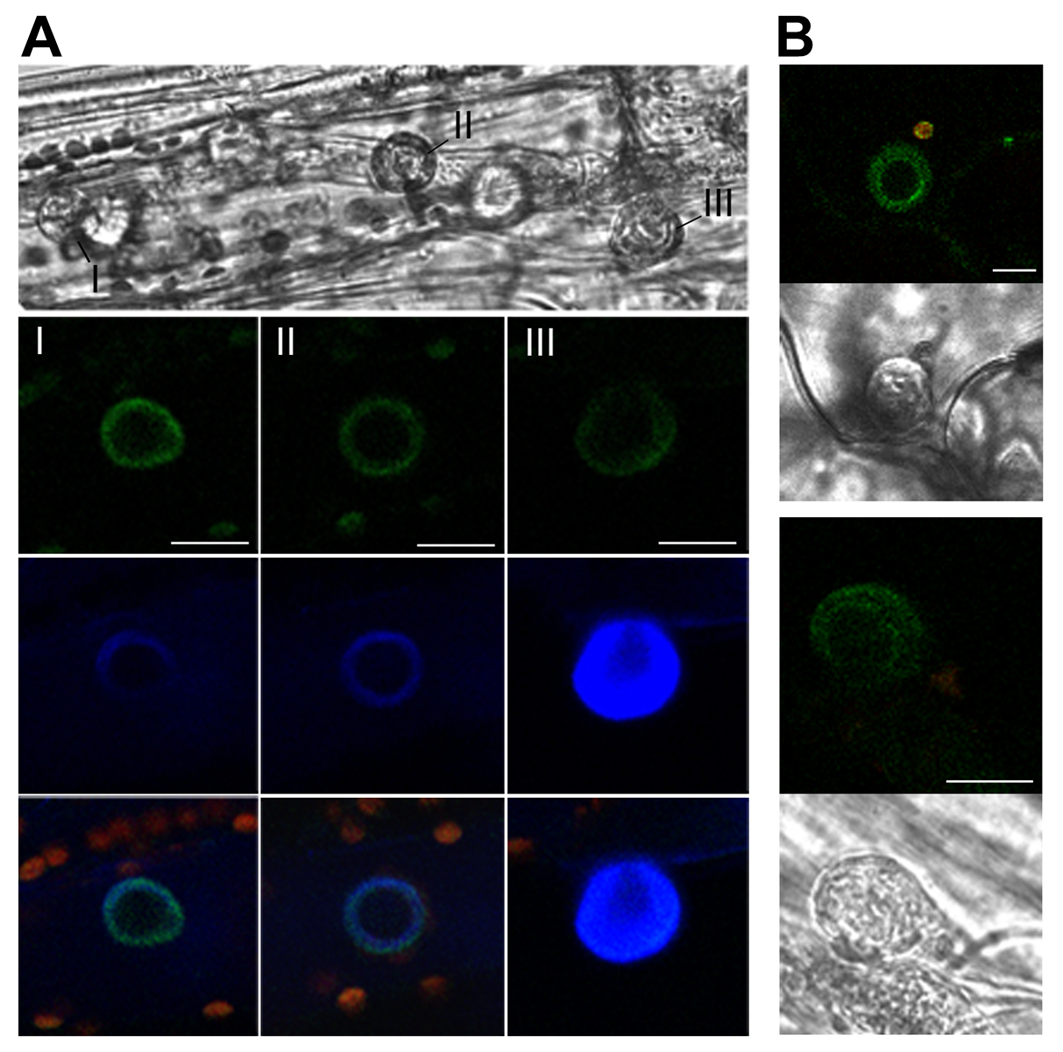

Supplement: Figure S2 — Haustorial association of PDLP1-GFP when expressed under the native promoter. (A) PDLP1-GFP (green) is present surrounding the haustorium before and during encasement. Haustoria (indicated as I, II and III in the brightfield image in the top panel) in varying stages of encasement each (aniline blue, blue) show PDLP1-GFP fluorescence surrounding the haustorium. Haustoria I–III show increasing levels of encasement. The bottom panel shows the overlay of PDLP1-GFP and aniline blue, red is chlorophyll autofluorescence. (B) In developing encasements PDLP1-GFP fluorescence can be resolved into two layers which are presumably membrane layers surrounding the encasement. Scale bars are 10 µm. (TIF) [file ppat.1004496.s002.tif]

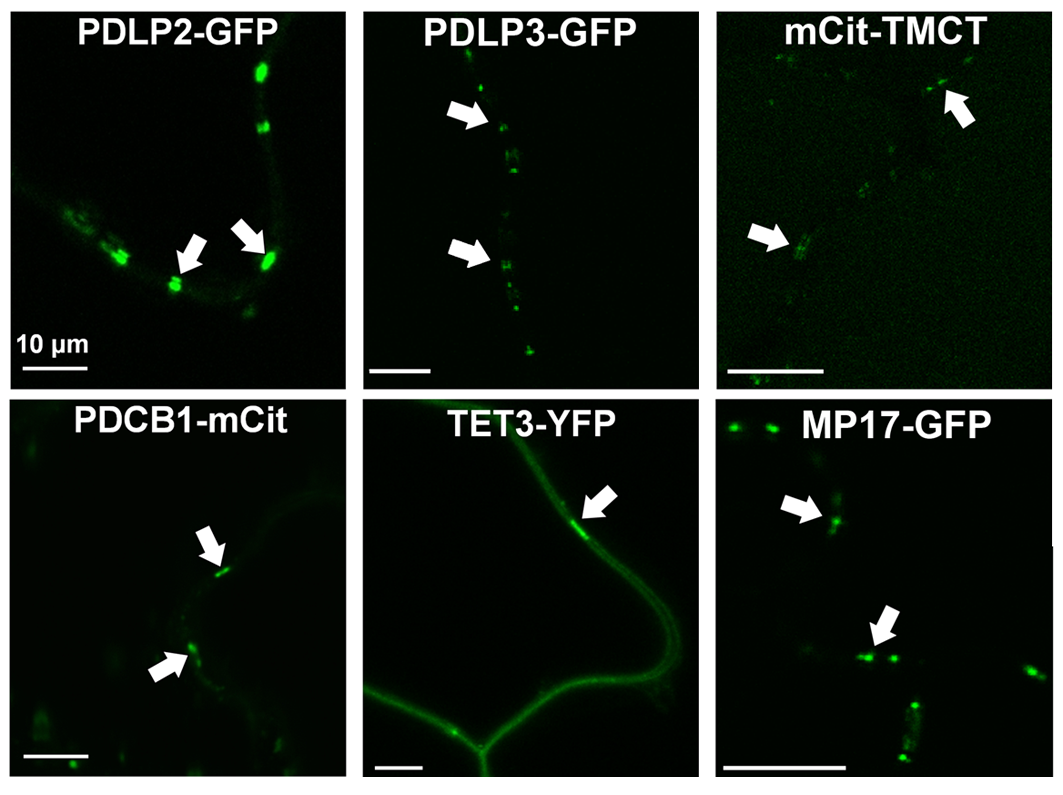

Supplement: Figure S3 — Subcellular localisation of the markers used in this study in non-infected cells. PDLP2-GFP, PDLP3-GFP, mCit-TMCT, PDCB1-mCit and MP17-GFP are all located at PD. TET3-YFP is visible in the PM and PD. PD are indicated by arrows, scale bars are 10 µm. (TIF) [file ppat.1004496.s003.tif]

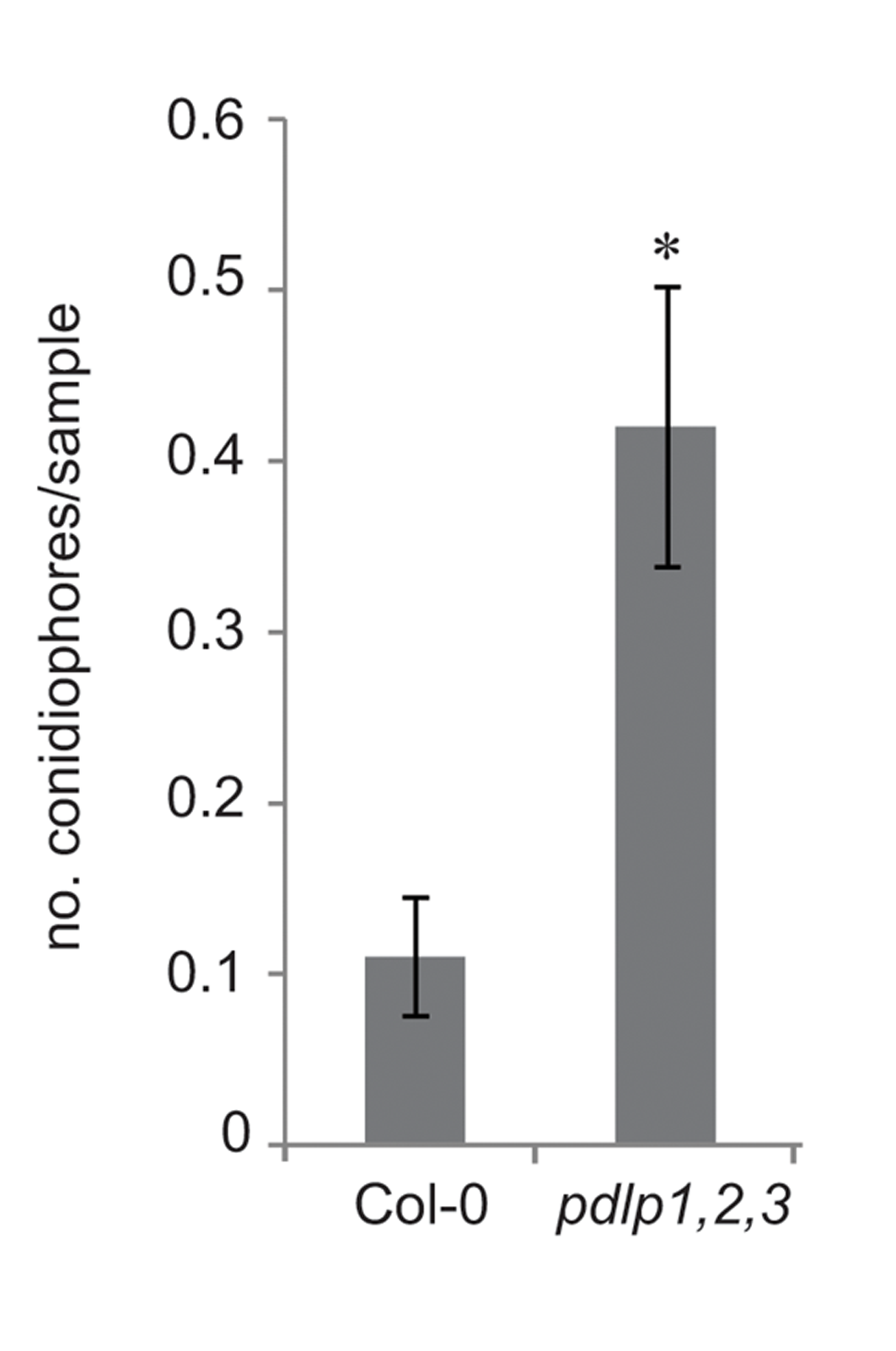

Supplement: Figure S4 — PDLPs positively regulate immunity to Hpa Emoy2. Conidiophore counting on cotyledons 6 DPI with Hpa Emoy2 on Col-0 and pdlp1,2,3 mutant plants. Error bars are the standard error of the mean, * indicates p-value <0.05 by non-parametric Mann-Whitney analysis. (TIF) [file ppat.1004496.s004.tif]

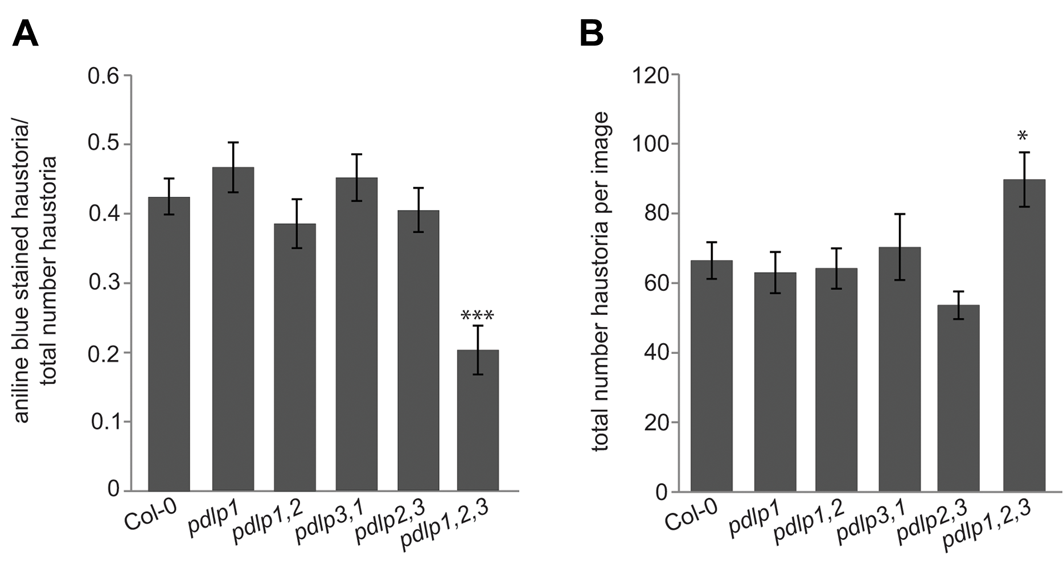

Supplement: Figure S5 — Reduced callose encasement in pdlp1,2,3 mutants is not due to reduced number of haustoria. (A) Number of haustoria (Hpa Noco) stained with aniline blue (encased haustoria) relative to the number of haustoria stained with trypan blue (total haustoria) for Col-0, the single knockout mutant pdlp1, the double knockout mutants pdlp1,2, pdlp2,3, pdlp3,1 and the triple knockout mutant pdlp1,2,3. Only the triple knockout pdlp1,2,3 shows a reduced proportion of encased haustoria. (B) The total number of haustoria per image for each genotype. pdlp1,2,3 mutant plants showed a greater number of haustoria per image, * indicates p-value <0.05 and *** indicates p-value <0.001 by Student's t-test. (TIF) [file ppat.1004496.s005.tif]

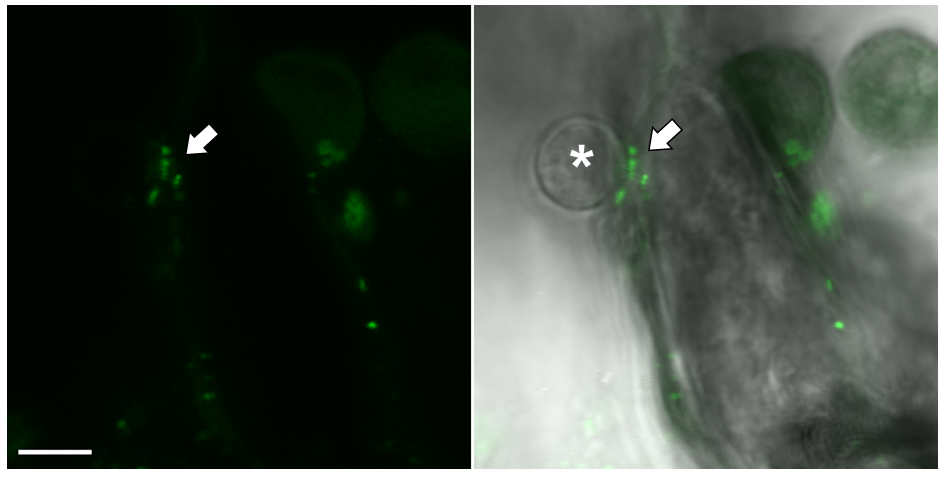

Supplement: Figure S6 — PDLP1-GFP does not localise to the EHM of Albugo laibachii haustoria. Some PDLP1-GFP fluorescence (left) is located at the neck of the haustorium but no label is seen around the periphery of the haustorium. The transmitted light image overlayed with the fluorescence image (right) identifies the position of the haustorium (asterisk). Scale bar is 5 µm. (TIF) [file ppat.1004496.s006.tif]

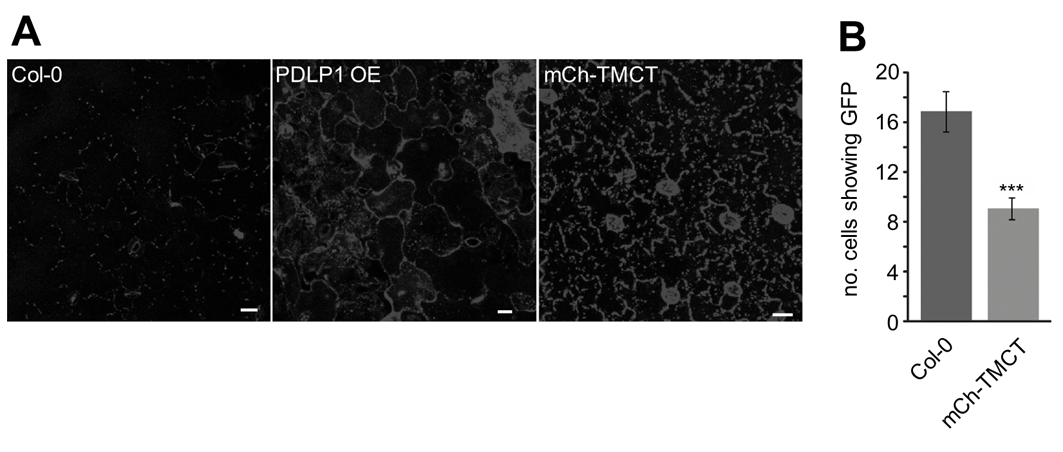

Supplement: Figure S7 — Overexpression of the PDLP1 TM and CT increases callose deposition at PD and reduces cell-to-cell flux. (A) Aniline blue staining of leaves of Col-0, PDLP1 OE and mCherry (mCh)-TMCT identifies that callose deposition is increased in both PDLP1 OE and mCh-TMCT plants relative to Col-0. In PDLP1 OE this staining is evident around the whole cell while in the mCh-TMCT leaves staining is discretely located at PD. Scale bars are 20 µm. (B) Microprojectile bombardment assays indicate that GFP diffusion from a bombardment site is reduced in mCh-TMCT leaves relative to Col-0. (TIF) [file ppat.1004496.s007.tif]
