# Supplementary material for: The Plasmodesmal Protein PDLP1 Localises to Haustoria-Associated Membranes during Downy Mildew Infection and Regulates Callose Deposition
Source: PLoS Pathog. 2014 Nov 13;10(11):e1004496. doi: 10.1371/journal.ppat.1004496 (PMC4231120; doi:10.1371/journal.ppat.1004496)
Supplement: Table S2 — Frequency and length of membrane invaginations observed by transmission electron microscopy. The total number of haustoria imaged by transmission electron microscopy for Col-0 and PDLPOE plants, and the frequency of membrane invaginations less than 100 nm and greater than 100 nm observed in these samples. (DOCX) [file ppat.1004496.s009.docx]

|  | No. of haustoria | No. with no invaginations | No. showing invaginations <100 nm | No. showing invaginations >100 nm |
| --- | --- | --- | --- | --- |
| Col-0 | 9 | 4 | 3 | 2 |
| PDLP1 OE | 9 | 0 | 3 | 6 |
