# Supplementary material for: The Plasmodesmal Protein PDLP1 Localises to Haustoria-Associated Membranes during Downy Mildew Infection and Regulates Callose Deposition
Source: PLoS Pathog. 2014 Nov 13;10(11):e1004496. doi: 10.1371/journal.ppat.1004496 (PMC4231120; doi:10.1371/journal.ppat.1004496)
Supplement: Methods S1 — Materials and methods used to generate data contained in the Supporting Information file. (DOCX) [file ppat.1004496.s010.docx]

**Methods S1**

**Callose staining of live tissue**

Leaves of 2-4 week old Arabidopsis plants were vacuum infiltrated with 0.1 % aniline blue and imaged by confocal microscopy.

**Co-staining with trypan blue and aniline blue**

Plants were spray inoculated with *Hpa* Noco as described and harvested 4-5 DPI. Aerial tissues were fixed and stained as described in [1]. Infected leaves were imaged with a Leica DM6000 epifluorescence microscope. Data was collected from three biological replicates for each genotype, and haustorial counts were made from at least 8 images per replicate.

**Microprojectile Bombardment**

4-6 week old expanded leaves of Arabidopsis were bombarded with 1 µm gold particles coated with pB7FWG2.0.GFP as described [2]. Bombarded leaves were imaged by confocal microscopy 20-24 h post bombardment.

**Pathoassays with *Albugo laibachii***

Plant inoculations were performed as described [3]. In brief, zoospores were diluted in water to 10^5^ spores/mL, incubated on ice for 30 min and spray inoculated on plants. Plants were incubated overnight in the dark at 4 ^o^C before being moved to short day (10 h light, 20 ^o^C; 16 h dark, 16 ^o^C) growth conditions.

**References**

1. Bhadauria V, Miraz P, Kennedy R, Banniza S, Wei Y (2010) Dual trypan-aniline blue fluorescence staining methods for studying fungus-plant interactions. Biotechnic & Histochem 85: 99-105.

2. Thomas CL, Bayer EM, Ritzenthaler C, Fernandez-Calvino L, Maule AJ (2008) Specific targeting of a plasmodesmal protein affecting cell-to-cell communication. PLoS Biol 6: e7.

3. Kemen E, Gardiner A, Schultz-Larsen T, Kemen AC, Balmuth AL, et al. (2011) Gene gain and loss during evolution of obligate parasitism in the white rust pathogen of *Arabidopsis thaliana*. PLoS Biol 9: e1001094.
